# Ribosomal protein gene *RPL9* variants can differentially impair ribosome function and cellular metabolism

**DOI:** 10.1093/nar/gkz1042

**Published:** 2019-12-04

**Authors:** Marco Lezzerini, Marianna Penzo, Marie-Françoise O’Donohue, Carolina Marques dos Santos Vieira, Manon Saby, Hyung L Elfrink, Illja J Diets, Anne-Marie Hesse, Yohann Couté, Marc Gastou, Alexandra Nin-Velez, Peter G J Nikkels, Alexandra N Olson, Evelien Zonneveld-Huijssoon, Marjolijn C J Jongmans, GuangJun Zhang, Michel van Weeghel, Riekelt H Houtkooper, Marcin W Wlodarski, Roland P Kuiper, Marc B Bierings, Jutte van der Werff ten Bosch, Thierry Leblanc, Lorenzo Montanaro, Jonathan D Dinman, Lydie Da Costa, Pierre-Emmanuel Gleizes, Alyson W MacInnes

**Affiliations:** 1 Amsterdam UMC, University of Amsterdam, Laboratory Genetic Metabolic Diseases, Amsterdam Gastroenterology and Metabolism, Meibergdreef 9, 1105 AZ Amsterdam, The Netherlands; 2 Laboratorio di Patologia Clinica, Dipartimento di Medicina Specialistica, Diagnostica e Sperimentale and Centro di Ricerca Biomedica Applicata (CRBA), Policlinico Universitario di S. Orsola, Università di Bologna,Via Massarenti 9, 40138 Bologna, Italy; 3 LBME, Centre de Biologie Intégrative (CBI), Université de Toulouse, CNRS, UPS, 31000 Toulouse, France; 4 Department of Cell Biology and Molecular Genetics, University of Maryland, College Park, MD, USA; 5 INSERM UMR S1134, F-75015, Paris, France; 6 Amsterdam UMC, University of Amsterdam, Laboratory Genetic Metabolic Diseases, Core Facility Metabolomics, Amsterdam Gastroenterology and Metabolism, Meibergdreef 9, 1105 AZ Amsterdam, The Netherlands; 7 Department of Human Genetics, Radboud University Medical Center, Nijmegen, The Netherlands; 8 University Grenoble Alpes, CEA, INSERM, IRIG, BGE, F-38000 Grenoble, France; 9 Paris University, Paris, France; 10 Laboratory of Excellence for Red Cell, LABEX GR-Ex, F-75015, Paris, France; 11 Institute Gustave Roussy, Inserm unit U1170, F-94800 Villejuif, France; 12 Department of Comparative Biology and Center for Cancer Research, Purdue University, West Lafayette, IN 47907, USA; 13 Department of Pathology, University Medical Center Utrecht, 3584 CX Utrecht, The Netherlands; 14 Department of Genetics, University Medical Center Utrecht, 3508 AB Utrecht, The Netherlands; 15 Department of Genetics, University of Groningen, University Medical Center Groningen, Groningen, The Netherlands; 16 Princess Maxima Center for Pediatric Oncology and Utrecht University Children's Hospital, Utrecht, The Netherlands; 17 Department of Pediatrics and Adolescent Medicine, Division of Pediatric Hematology and Oncology, Medical Center, Faculty of Medicine, University of Freiburg, D-79106 Freiburg, Germany; 18 St. Jude's Children Research Hospital, Memphis, TN, USA; 19 Department of Pediatrics, Universitair Ziekenhuis Brussel, Brussels, Belgium; 20 Pediatric Hematology/Oncology Service, Robert Debré Hospital, F-75019 Paris, France; 21 Hematology Lab, Robert Debré Hospital, F-75019 Paris, France

## Abstract

Variants in ribosomal protein (RP) genes drive Diamond-Blackfan anemia (DBA), a bone marrow failure syndrome that can also predispose individuals to cancer. Inherited and sporadic RP gene variants are also linked to a variety of phenotypes, including malignancy, in individuals with no anemia. Here we report an individual diagnosed with DBA carrying a variant in the 5′UTR of *RPL9* (uL6). Additionally, we report two individuals from a family with multiple cancer incidences carrying a *RPL9* missense variant. Analysis of cells from these individuals reveals that despite the variants both driving pre-rRNA processing defects and 80S monosome reduction, the downstream effects are remarkably different. Cells carrying the 5′UTR variant stabilize TP53 and impair the growth and differentiation of erythroid cells. In contrast, ribosomes incorporating the missense variant erroneously read through UAG and UGA stop codons of mRNAs. Metabolic profiles of cells carrying the 5′UTR variant reveal an increased metabolism of amino acids and a switch from glycolysis to gluconeogenesis while those of cells carrying the missense variant reveal a depletion of nucleotide pools. These findings indicate that variants in the same RP gene can drive similar ribosome biogenesis defects yet still have markedly different downstream consequences and clinical impacts.

## INTRODUCTION

Diamond-Blackfan anemia (DBA) (OMIM# 105650) is an inherited bone marrow failure disorder that typically presents in children less than one year of age. While the central phenotype is pure red cell aplasia and a paucity of erythroblast precursor cells in the bone marrow, a number of physical malformations are also linked to DBA (1). These include (but are not limited to) craniofacial malformations, growth retardation, abnormalities in the extremities (especially the thumb), heart defects, and urogenital defects (2,3). DBA patients also have an elevated cancer risk, particularly hematologic malignancies, osteosarcoma, and colon carcinoma (4,5).

With rare exceptions, DBA is a disease linked to RP gene variants (6). These RPs include eS7 (*RPS7*), uS8 (*RPS15A*), eS10 (*RPS10*), eS17 (*RPS17*), eS19 (*RPS19*), eS24 (*RPS24*), eS26 (*RPS26*), eS27 (*RPS27*), eS28 (*RPS28*), uS14 (*RPS29*), uL18 (*RPL5*), uL5 (*RPL11*), eL15 (*RPL15*), eL18 (*RPL18*), uL24 (*RPL26*), eL27 (*RPL27*), eL31 (*RPL31*), uL29 (*RPL35*), eL33 (*RPL35A*) (7–21), and a phenotype resembling DBA has been identified in one individual carrying a variant in uL4 (*RPL4*) (22). DBA mutations are heterozygous and result in haploinsufficiency of the corresponding RP, which affects processing of the pre-ribosomal RNA (pre-rRNA) in a RP-specific manner. *RPL9* gene allelic variation has so far been reported in one DBA-affected individual, however this c.375G>C; p.Arg125Ser variation was declared to be a variant of unknown significance (VUS) since cells from this patient did not show a pre-rRNA processing defect similar to that observed upon knockdown of RPL9 with siRNAs (9). Although the pathophysiology linking RP variants to the DBA bone marrow failure phenotype is not entirely understood, the stabilization of the TP53 tumor suppressor protein is thought to occur due to ribosomal stress and in turn plays a role in impairing the proliferation of CD34^+^ erythroblast precursor cells (23–25). In fact, a recent study reported germinal *TP53* gene activating variants in two individuals with a DBA-like phenotype that includes erythroblastopenia (26).

An increasing number of RP genes carrying inherited or sporadic variants are being uncovered that do not drive the bone marrow failure that is the hallmark of DBA. Missense variants in *RPS23* (OMIM #617412) and *RPL10* (OMIM #300847 and #300998) are found in individuals with dysmorphism, autism, and intellectual disability who have no evidence of a hematological phenotype (27–30). Somatic variants in RP genes have also been found in several cancer exomes. These include acute lymphoblastic T-cell leukemia (T-ALL) (*RPL10* and *RPL5*); glioma, melanoma, and breast cancer (*RPL5*); colorectal and endometrial cancer (*RPL22*); and chronic lymphocytic leukemia (*RPS15*) (31–34). Inherited variants in *RPS20* have also been reported linked to hereditary nonpolyposis colon carcinoma (OMIM #120435) (35). Although none of these variants have been shown to drive stabilization of TP53, the *RPS23* p.Arg67Lys variant linked to dysmorphism and the *RPL10* p.Arg98Ser variant linked to T-ALL are reported to alter the translational fidelity of ribosomes by increasing frameshifting and the readthrough of stop codons (28,36). Interestingly, despite not driving an anemia phenotype and having no observed effect on TP53, the missense variants in *RPS23* p.Arg67Lys and *RPL10* p.Arg98Ser have been reported to impair the processing of pre-rRNA and affect the formation of polysomes (28,37). Thus, it appears that variants in RPs that impair ribosome biogenesis do not universally drive anemia and that the clinical phenotypes linked to the variants are dependent on a more complex set of events.

Here, we report that different variants in *RPL9*, a gene that has not been definitively described in DBA or other human diseases, drive similar defects in the processing of pre-rRNA during ribosome biogenesis yet reveal markedly different downstream effects on the TP53 pathway, erythrocyte development, metabolism, and the ability of ribosomes to recognize mRNA stop codons. This study endeavors to unravel the similarities and differences between variants found in the same RP gene and how they might ultimately contribute to the resulting clinical phenotypes.

## MATERIALS AND METHODS

### Patients

One individual was identified within the European DBA (EuroDBA) consortium registries. The diagnosis of DBA in this individual was established based on typical features including aregenerative anemia with erythroid hypoplasia (1). Two other individuals (mother and son) were identified in a study on genetic predisposition for childhood cancer. Written informed consent was obtained from patients and/or parents prior to inclusion in this study, which was performed in accordance with the ethical standards of the Declaration of Helsinki. All procedures herein were performed according to the standards of the institutional and national ethical boards.

### Cell culture and treatment with siRNA duplexes

Lymphoblastoid cell lines (LCLs) were derived from EBV-immortalization of peripheral mononuclear cells isolated from whole blood using Ficoll (GE Life Sciences) and grown in RPMI (Gibco) containing 10% fetal calf serum (FCS), 1% l-glutamine, and 1% penicillin/streptomycin as previously described (38). HeLa cells were cultured in DMEM (Gibco) supplemented with 10% fetal calf serum and 1 mM sodium pyruvate (Sigma). Two 19-mer siRNAs (Eurogentec), whose efficiency was verified by qPCR, were used to knock down expression of human uL6 mRNAs in HeLa cells: si-uL6-1 (5′-CCCAGAAAGAUGAAUUAAUdTdT-3′) and si-uL6-2 (5′-GGGACUUCAAUCACAUCAAdTdT-3′). Each siRNA solution was added at a final concentration of 500 nM to 10^7^ cells diluted in sodium phosphate buffer, pH 7.25, containing 250 mM sucrose and 1 mM MgCl_2_. Electro-transformation was performed at 240 V with a Gene Pulser (Bio-Rad) (39). Control HeLa cells were electro-transformed with a scramble siRNA (siRNA-negative control duplex; Eurogentec). After 10 min incubation at ambient temperature, cells were plated and grown at 37°C for 48 h.

### Sanger sequencing of LCLs

mRNA from LCLs was isolated with Trizol (ThermoFisher) for generation of cDNA using the QuantiTect Reverse Transcription Kit 400 (Qiagen) per the manufacturers’ instructions. Primers used for Sanger sequencing of the 5′ UTR variant are FW:5′-TTCTCAGCAATCAGACTGTCG-3′ and REV:5′-AGGCTGGTATTCTGGACAGC-3′. Primers used for sequencing the p.Leu20Pro variant are FW:5′-CGGGAAAGGACAGGTCGAAA-3′ and REV:5′-GACAAGGTTCCGAGAGTGGG-3′.

### Genomic studies and bioinformatics

Whole exome sequencing (WES) was performed using SOLiDv4 (Life Technologies) or HiSeq2500 (Ilumina) sequencers after enrichment with the Agilent SureSelectXT Human All Exon 50Mb kit. Data analysis was done with BioScopeTM and pathogenicity calculations were performed using standard prediction tools. For P3, both tumors (osteosarcoma and AML) were sequenced, as germline material derived from blood was not available due to a hematopoietic cell transplantation (HCT). Specific consent for making the exome sequencing data public was not granted. However, any questions regarding the data may be directed to the clinical geneticist, Marojolijn Jongmans, at M.C.J.Jongmans-3@umcutrecht.nl. Variants that were present in both tumors and in one of the parents were considered to be germline variants. The analysis focused on variants present in both tumors of the index patient (P3) and his affected mother (P2). Normal variation, defined as variants present in >10 cases of our in-house database and variants present with a frequency of more than 1% in dbSNP [build 144 (40)], the ExAC or the gnomAD database (41) were excluded. Missense variants were filtered based on Call quality (≥10 total reads with ≥20% variant reads), conservation score (PhyloP ≥ 3.0) and pathogenic prediction in two out of three computational prediction scores CADD PHRED (42) (version 1.3, >20), SIFT (43) (damaging) and PolyPhen2 (44) (possibly/probably damaging) using Alamut Visual software (v2.7.1., Interactive Biosoftware, Rouen, France). Multiple sequences alignments were performed using Clustal Omega online software (https://www.ebi.ac.uk/Tools/msa/clustalo).

### Total RNA extraction and analysis of pre-rRNA processing by northern blot

Total RNAs were extracted with Trizol from cell pellets containing 20 × 10^6^ cells. The aqueous phase was further extracted with phenol-chloroform-isoamylic alcohol (25:24:1; Sigma), then with chloroform. Total RNAs were recovered after precipitation with 2-propanol. For Northern blot analyses, RNAs were dissolved in formamide, denatured for 10 min at 70°C and separated on a 1.2% agarose gel containing 1.2% formaldehyde and 1× Tri/Tri buffer (30 mM triethanolamine, 30 mM tricine, pH 7.9) (3 μg RNAs/lane). RNAs were transferred to a Hybond N^+^ nylon membrane (GE Healthcare) by passive transfer. Pre-hybridization was performed for 1 h at 45°C in 6× SSC, 5× Denhardt's solution, 0.5% SDS, 0.9 g/ml tRNA. The 5′-radiolabeled oligonucleotide probe was incubated overnight. The sequences of the probes were: 5′ITS1 (5′-CCTCGCCCTCCGGGCTCCGTTAATGATC-3′), ITS1-5.8S (5′-CTAAGAGTCGTACGAGGTCG-3′), ITS2 (ITS2b: 5′-CTGCGAGGGAACCCCCAGCCGCGCA-3′ and ITS2d/e: 5′-GCGCGACGGCGGACGACACCGCGGCGTC-3′), 18S (5′-TTTACTTCCTCTAGATAGTCAAGTTCGACC-3′), 28S (5′-CCCGTTCCCTTGGCTGTGGTTTCGCTAGATA-3′). Membranes were washed twice for 10 min in 2× SSC, 0.1% SDS and once in 1× SSC, 0.1% SDS, and then exposed. Signals were acquired with a Typhoon Trio PhosphoImager (GE Healthcare) and quantified using the MultiGauge software.

### Polysome profiling analysis

600 μg to 1 mg of total proteins from freshly lysed LCLs was loaded onto 10–50% sucrose gradients as previously described (45). The tubes were centrifuged at 4°C and at 36 000 rpm for 2 h in a SW41 rotor (Optima L100XP ultracentrifuge; Beckman Coulter). The gradient fractions were measured at OD_254nm_ using a syringe pump and UV detector (Brandel) and collected with a Foxy Jr gradient collector (Teledyne Isco).

### Western blot analysis

For TP53 stabilization, LCLs were plated at 100 000 per well of a six-well dish overnight and camptothecin (100nM in DMSO) (Sigma) or an equal volume of DMSO as a vehicle control was added to wells for 6 h before lysis in RIPA buffer with proteasome inhibitors (Sigma). 10% acrylamide gels were run for detection of TP53 (Santa Cruz #sc-6243) and actin (Santa Cruz #sc-1616).

### Zebrafish mutant strains and *o*-dianisidine staining

Zebrafish hi1422 (*rpL9*) mutants were created in the lab of Dr Nancy Hopkins and maintained as described (46). Matings were performed between two adult heterozygous hi1422 fish. At 2 days post fertilization (dpf) embryos were stained with *o*-dianisidine (Sigma) as previously described (47). Scoring of the phenotype severity was done blindly. Embryos from the hi1422 mating were genotyped after staining using Sanger sequencing with primers 1422–5: 5′-ACCTGGAACTCAGTCTGTTGG-3′; hi14223R1: 5′-GCTTGCAGGCAGAGTGGGAG-3′; MSL4 (viral): 5′-GCTAGCTTGCCAAACCTACAGGT-3′. 3-primer PCR conditions were 95°C for 3 min; (95°C 30′ s, 55°C 30 s, 72°C 1 min) × 35; 72°C 5 min; 12°C.

### Erythroid cell culture assays

Erythroid cell culture assays were performed as previously described (25,48). Antibodies and stains for FACS analysis were PC7 or PE conjugated CD34 (Beckman coulter, Brea, CA, USA), APC conjugated CD36 (BD Biosciences San Jose, CA, USA), APC conjugated Band 3 (kindly provided by Mohandas Narla's lab, NYBC, New York, USA), PE/Cy7 conjugated IL-3R (Miltenny, Paris, France), PE/Cy7 (PE)-coupled GPA (Life Technologies Carlsbad, CA, USA), and DAPI (Sigma). Antibodies for western blotting were directed against TP53 (Sigma #5816), CDKN1A/p21 (Cell Signaling #2947), actin (Sigma #Ac-15), and uL6 (Abcam #ab102011). FACS analysis was conducted on a BD Biosciences Influx flow cytometer (BD Biosciences San Jose, CA, USA). Data were analyzed using Kaluza software (Beckman coulter, Brea, CA, USA).

### Immunohistochemistry

Colon tissue was biopsied and slides stained by standard Haemotoxylin and Eosin (H&E) staining for morphology. Antibodies used for staining were against TP53 (Dako #M7001) and CD3 (Dako #A0452).

### Translational fidelity reporters

For the cell-based assays a new generation of dual luciferase reporters was generated by Loughran *et al.* to correct interference of test sequence with stability and activity of *Renilla* and firefly luciferases (49). Plasmid pSGDluc, which contains tandem StopGo sequences (2A) on either side of the test sequence (49), was kindly provided by Dr John Atkins, at University College Cork. In order to disrupt the *BamHI/SalI* sites present downstream of the firefly luciferase coding sequence, complimentary oligonucleotides (BamSalKilT and BamSalKilB, sequences available upon request) were ligated with linearized vector. The resulting plasmid was doubly digested with *XhoI* and *ApaI*, and the vector portion was used in Gibson assembly. A gBlock (sequences available upon request) was used to insert the HIV -1 PRF signal (50,51) between new *SalI* and *BamHI* sites. After sequence verification, the resulting plasmid (pJD2256) was linearized with *BamHI* and *SalI*, and another set of complementary oligonucleotides (Alphahelixspacer-T2 and Alphahelixspacer-B2, sequences available upon request) was used to insert an alpha helix spacer which creates an open reading frame encoding the peptide sequence: EAAAKEAAAKA. The resulting plasmid, pJD2257, was used as the zero frame dual luciferase reporter. pJD2337 (PEG10) was used to monitor −1 PRF, and pJD2349 (OAZ1) was used to monitor +1 PRF. pJD2443 (UAA), pJD2444 (UGA) and pJD2445 (UAG) were used to monitor stop codon readthrough. To make the constructs used to monitor PEG10 (52,53) and OAZ1 (54) mediated −1 and +1 PRF, gBlocks were inserted in linearized pJD2257 by Gibson Assembly (sequences available upon request). To make the constructs used to monitor stop codon readthrough, sets of complimentary oligonucleotides (sequences available upon request) were ligated with linearized pJD2257.

### Translational fidelity cell-based assays

Deidentified LCLs were transfected by electroporation using an Amaxa™ Nucleofector II apparatus and Cell Line Nucleofector^®^ Kit V (Lonza), per the manufacturer's instructions. For each transfection, 1.5 × 10^6^ cells were mixed with 1.5 μg of plasmid DNA. All assays were performed in triplicate four independent times. Cell lysates were prepared using passive lysis buffer and luciferase activities were determined using the Dual-Luciferase^®^ Reporter Assay System and a GloMax-Multi+ Detection System (Promega), per the manufacturer's instructions 24 hours post-transfection. Data were analyzed by dividing the ratio of firefly luciferase to *Renilla* luciferase for each of the experimental plasmids by the same ratio for the readthrough plasmid control in the same experiment. Data were plotted on GraphPad Prism as percent translational recoding, with each symbol representing one biological sample assayed in triplicate. Error bars represent standard deviation. Normal distribution of the data was determined by the Shapiro–Wilk normality test. Statistical significance was obtained by one-way ANOVA, followed by Holm–Sidak's multiple comparisons test. Adjusted *P*-values were reported on graphs as *P < 0.05 and **P < 0.01.

### Translational fidelity cell-free assays

Cell-free assessment of ribosome fidelity was performed essentially as previously described (55,56). Briefly, cytoplasmic cellular lysates were prepared from subconfluent LCLs. From these lysates, ribosomes were purified in stringent conditions on a discontinuous sucrose gradient by a 15–16 h centrifugation at 160 000g. To test STOP codon readthrough, assays were performed for 60 min at 30°C in reaction mixtures containing 30 mM HEPES/KOH, pH 7.5, 80 mM KCl, 1.8 mM magnesium acetate, 50 μM of each amino acid, 1 mM ATP, 0.25 mM GTP, 5 mM creatine phosphate, 0.18 mg/ml creatine phosphokinase, 0.5 mM DTT, 0.4 mM spermidine, S-140 (40% of reaction volume), ribosomal salt wash (4% of reaction volume), 300 ng of capped reporter mRNA [wild type (WT) or STOP mutant firefly luciferase (FLuc) mRNAs were mixed with an mRNA coding for Renilla luciferase (RLuc), as an internal reference, in a 49:1 ratio], and 0.125 pmol ribosomes. After incubation time, the reactions were stopped on ice and RLuc and FLuc activities were measured using a Dual-Luciferase Reporter assay (Promega Corp.), following manufacturer's specifications. To calculate the relative STOP codon readthrough (expressed in %), FLuc/RLuc ratios were calculated for each sample, after background subtraction. Then, for each ribosome type, the STOP mutant FLuc/Rluc ratio was expressed as percentage of WT FLuc/RLuc ratio. Subsequently, the ratios obtained for mutant samples were normalized on the averaged ratios obtained for WT samples.

### Mass spectrometry (MS)-based proteomic analyses

Proteins from cell-free ribosomal preparations were solubilized in Laemmli buffer before being stacked in the top of a 4–12% NuPAGE gel (Life Technologies), stained with R-250 Coomassie blue (Bio-Rad) and in-gel digested using modified trypsin (sequencing grade, Promega) as previously described (57).

The dried extracted peptides were resuspended in 5% acetonitrile and 0.1% trifluoroacetic acid and analyzed by online nanoliquid chromatography coupled to tandem mass spectrometry (LC–MS/MS) (Ultimate 3000 RSLCnano and the Q-Exactive HF, Thermo Fisher Scientific). Peptides were sampled on a 300 μm 5mm PepMap C18 precolumn (Thermo Fisher Scientific) and separated on a 75 μm 250 mm C18 column (Reprosil-Pur 120 C18-AQ, 1.9 μm, Dr. Maisch HPLC GmbH). The nano-LC method consisted of a 120 min multi-linear gradient at a flow rate of 300 nl/min, ranging from 5 to 33% acetonitrile in 0.1% formic acid. The spray voltage was set at 2 kV and the heated capillary was adjusted to 270°C. Survey full-scan MS spectra (*m*/*z* = 400–1600) were acquired with a resolution of 60 000 after the accumulation of 10^6^ ions (maximum filling time 200 ms). The 20 most intense ions were fragmented by higher-energy collisional dissociation after the accumulation of 10^5^ ions (maximum filling time: 50 ms). MS and MS/MS data were acquired using the software Xcalibur (Thermo Scientific).

Data were processed automatically using Mascot Distiller software (version 2.7.1.0, Matrix Science). Peptides and proteins were identified using Mascot (version 2.6) through concomitant searches against Uniprot (Homo sapiens taxonomy, June 2019 version), the mutated RPL9 protein sequence, classical contaminants database (homemade) and their corresponding reversed databases. Trypsin/P was chosen as the enzyme and two missed cleavages were allowed. Precursor and fragment mass error tolerance were set, respectively, to 20 ppm and 25 mmu. Peptide modifications allowed during the search were: carbamidomethylation (fixed), acetyl (protein N-terminal, variable) and oxidation (variable). The Proline software (http://proline.profiproteomics.fr) was used to filter the merged results: conservation of rank 1 peptide-spectrum match (PSM) with a minimal length of 7 and a minimal score of 25. PSM score filtering is then optimized to reach a False Discovery Rate (FDR) of PSM identification below 1% by employing the target decoy approach. A minimum of one specific peptide per identified protein group was set. Proline was then used to perform MS1-based label free quantification of the peptides and protein groups from the different samples. The mass spectrometry proteomics data have been deposited to the ProteomeXchange Consortium via the PRIDE partner repository with the dataset identifier PXD015218 (58).

### uL6 structure modeling

Modeling of wild type uL6 structure compared to uL6 p.Leu20Pro was performed in Pymol using the mutagenesis function. Source file for this was RCSB PDB entry 3J7P, the structure of the 80S mammalian ribosome bound to eEF2 (59).

### Metabolic profiling

For the cell culturing and liquid extraction: LCLs were seeded and cultured 24 h before harvesting at an appropriate density for logarithmic cell growth. Cells were washed 0.9% (w/v) NaCl (Fresenius Kabi, Germany) and harvested in 1 ml ice-cold 5/5 (v/v) methanol (Biosolve, The Netherlands)—water, transferred to 2 ml safe-lock Eppendorf (Germany) tubes. Internal standards D_3_-alanine, D_3_-aspertate, D_3_-arginine, D_3_-proline, D_3_-leucine, ^13^C_1_-citruline, D_3_-methionine, D_3_-aspartic acid, D_4_-tyrosine, D_4_-lysine, D_8_-valine, D_6_-ornithine, D_3_-serine, D_5_-tryptophan, D_5_-glutamine, D_5_-phenylalanine, D_3_-glutamate, ^13^C_3_-pyruvate, ^13^C_6_-isoleucine, D_6_-Succinate, ^13^C_6_-glucose, ^13^C_6_-fructose-1,6-biphosphate, ^13^C_6_-glucose-6-phosphate, adenosine-^15^N_5_-monophosphate, adenosine-^15^N_5_-triphosphate and guanosine-^15^N_5_-monophosphate and guanosine-^15^N_5_-triphosphate (5 μM) were added to the homogenates prior to the addition of 1 ml chloroform (Biosolve). The samples were vortexed and centrifuged at 14 000 rpm at 4°C. The aqueous upper-phase was collected and evaporated to dryness in a vacuum concentrator. The dried polar metabolites were reconstituted in 100 μl 6/4 (v/v) methanol–water. For the liquid chromatography and mass spectrometry, the analytical separation was performed on a Dionex Ultimate 3000 ultra-high performance liquid chromatography system (Thermo Fisher Scientific, USA) with a SeQuant Zic-cHILIC, 3 μm, 100 Å, 100 × 2.1 mm PEEK coated HPLC column (1.50657.0001, Millipore, USA) kept at 15°C. Mobile phase A consisted of 5 mM ammonium acetate (Sigma-Aldrich, USA) in acetonitrile (Biosolve)–water (9:1 v/v) and mobile phase B consisted of 5 mM ammonium acetate in acetonitrile–water (1:9 v/v). The flow of the 35 min gradient was 0.25 or 0.4 ml/min, as explained below, and ran from 0% to 100% B. The initial equilibration ran at a flow of 0.25 ml/min at 0% B lasts 2 min. The linear gradient runs from 0% to 100% B between 2–28 min. The hold period at 100% B was maintained from 28 to 30 min and from 30 to 31 min the mobile phase was changed back to 0% B. Re-equilibration at 0% B is from 32–35 min with an increased flow of 0.4 ml/min. The injection volume was 5 μl. The analysis was performed on a Q Exactive Plus Orbitrap mass spectrometer (Thermo Fisher Scientific) with a HESI source with a spray voltage of 2.5 kV, a capillary temperature of 253°C, and the S-lens RF level set at 50.0. All the (remaining) source parameters were optimized using the automated algorithm from the Q Exactive Plus software (Exactive series slot #1, version 2.9 Build 2926). Data analysis was performed using Thermo Scientific Xcalibur software (Version 4.1.50, Build date Monday, 9 October 2017), and processed using in house developed statistical package in R (version 3.3.1, 2016-06-21). Enrichment analysis was performed with MetaboAnalyst 3.0 online software (https://www.metaboanalyst.ca).

## RESULTS

### Gene variants in *RPL9* link to DBA and multiple cancer incidences

**Figure 1.**
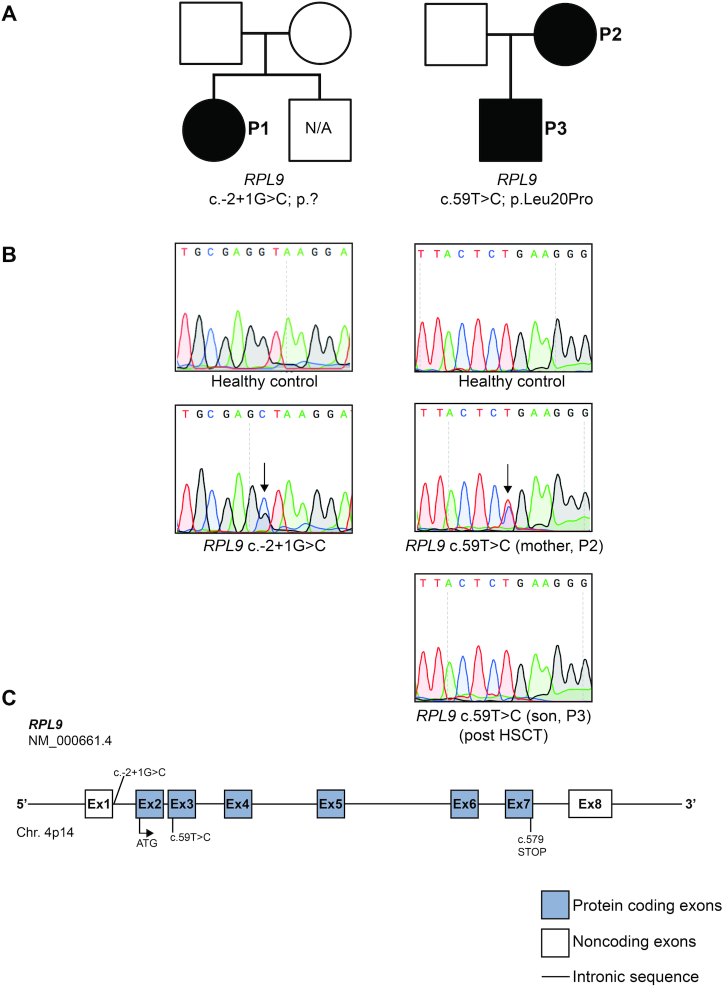
Heterozygous variants in *RPL9* are linked to human disease. (**A**) Two pedigrees of individuals affected by DBA (left) or multiple cancer incidences (right). Affected variant carriers are indicated with filled squares (male) or circles (female) and are identified as Patients 1–3 (P1–4). Unaffected individuals are indicated by unfilled symbols. N/A indicates unaffected family members who were not investigated for the presence of variants. (**B**) Sanger sequencing results of LCLs derived from heathy controls or the affected individuals in (A). The double peaks illustrating variant allele expression are indicated with arrows. (**C**) Schematic representation of the human *RPL9* gene depicting localization of the variants identified in the families in (A).

Whole exome sequencing (WES) of patient-parent trios identified an individual within the EuroDBA consortium registries with the heterozygous variant c.-2+1G>C (P1) in the 5′UTR of the *RPL9* gene (NM_000661.4) (Table 1 and Figure 1). The c.-2+1G>C variant appears to be *de novo* as both parents tested wild-type for *RPL9*. Sanger sequencing of cDNA from EBV-immortalized lymphoblast cell lines (LCLs) derived from these individuals confirmed the presence of double peaks, indicating that the variant allele is expressed (Figure 1B). The c.-2G+1>C variant is predicted to be a splice site variation affecting the donor splice site of exon 1 (Figure 1C) and has previously not been reported in SNP databases. This individual, in addition to anemia, presented with an abnormal thumb and colitis, the pathology of which was marked by apoptotic bodies, CD3^+^ lymphocyte infiltration, and TP53 stabilization (Table 1 and Supplementary Figure S1).

**Table 1.** Clinical features of individuals carrying variants in RPL9

Patient;sex	RP gene variant	Clinical presentation and therapies	Gestational age; malformations; other	Age and status at last follow-up	Family History
1;F	*RPL9* c.-2+1G>C;p.?	DBA onset: 6 months old; Hb 8.7 g/dL Lab: MCV/eADA normal HbF↑(1.7%) Evolution: 2 failed steroid courses. Transfusion-dependent for 1 year, thereafter fewer transfusions required.	37 weeks, microcephaly, colitis, thumb anomaly (1 side afunctional, other side finger- like), failure to thrive. Autoimmune colitis at age 6 months, received steroids and azathioprine.	4 years, irregular transfusions.	Parents wild type. Two miscarriages, one healthy newborn brother.
2;F	*RPL9* c.59T>C; p.Leu20Pro	Squamous cell carcinoma of the vulva diagnosed at age 43. Tumor was surgically resected.	Short stature (length 155cm, -2SD). Tested negative for HPV.		Mother of P3
3;M	*RPL9* c.59T>C; p.Leu20Pro	Conventional type osteosarcoma diagnosed at age 11. Chemotherapy according to the EURAMOS-1 protocol (good responder arm: 4x MAP, 2x MAPEI). AML subtype M5 diagnosed at age 13. Treated with induction- and consolidation chemotherapy according to the NOPHO-DHB AML 2012 protocol, and a subsequent allogeneic stem cell transplantation from a matched-unrelated donor (MUD).	Micropenis and short stature (length 165cm, −2.25SD).		Son of P2

Another family carrying a *RPL9* variant was identified by WES within a study on childhood cancer predisposition [methods described in (60)]. This family consisted of a boy (P3) diagnosed with childhood cancer twice, and his mother (P2). P3 presented with conventional type osteosarcoma (Table 1). Physical examination revealed a micropenis, short stature (-2.25SD), and no dysmorphic features. He was treated with chemotherapy according to the EURAMOS-1 protocol (good responder arm: 4× MAP, 2× MAPEI). Less than two years later at age 13, he developed acute myeloid leukemia (AML), subtype M5. This AML may have been secondary to the chemotherapy received for the osteosarcoma. However, this possibility was considered not likely given the short period of time between the chemotherapy and the AML, the fact that the karyotype was normal, the patient did not receive etoposide, and because the AML was very treatable (remission occurred very quickly, which is unusual with a typically aggressive secondary AML). The AML was treated with induction and consolidation chemotherapy according to the NOPHO-DHB AML 2012 protocol, and a subsequent HCT from a matched-unrelated donor (MUD). Family history showed that his mother (P2) was diagnosed with a squamous cell carcinoma of the vulva at age 43 and that she had tested negative for the human papilloma virus (HPV). She had no congenital anomalies or dysmorphic features, but had short stature as well (length 155 cm, −2SD) which is a feature that is commonly reported in DBA-affected individuals (61).

WES was performed on germline DNA of both parents of P3, and tumor-derived DNA from both the osteosarcoma and the AML. No germline material of P3 could be obtained, since he had been HCT-treated. A missense variant in *RPL9* c.59T>C; p.Leu20Pro was identified in both types of tumors of P3, which was maternally inherited (from P2) and occurs in exon 3 (Figure 1C and Supplementary Figure S2). Given that this variant appeared in both tumors and in one parent of P3, we consider it a germline mutation. This Leu20 residue precedes a universally conserved residue in uL6 (Lys21), and is itself highly conserved in mammals, birds, frogs, and fish (Supplementary Figure S3). Importantly, this residue preceding Lys21 is invariably hydrophobic, as it is found as isoleucine or valine in flies, worms, and yeast (Supplementary Figure S3). No loss of heterozygosity or second hit mutations in *RPL9* in either of the tumors of P3 was present. Moreover, the exome sequencing of the two tumors revealed variants that were not present in either parents and as such considered to be somatic (Supplementary Table S1), however none of these variants to date have any reported links to cancer. Sanger sequencing of LCLs derived from the mother (P2) revealed a double peak which was not present in the son (P3) given the LCLs were derived post-HCT (Figure 1B). This variant has been previously reported (rs141176319) at a very low frequency of 0.00005767. While it may be that some individuals carrying the variant are unaffected due to decreased penetrance of the variant, it is also possible that these individuals may be predisposed to developing cancer later in life. Another possibility is that p.Leu20Pro is a VUS, although the following functional data suggest that this variant does have a detrimental effect on ribosome biogenesis resembling pathogenic DBA-linked RP gene variants.

### *RPL9* variants impair the processing of pre-rRNAs

The normal processing and maturation of pre-rRNAs in humans, illustrated in Supplementary Figure S4, is impaired at different steps by DBA-linked RP gene variants (9,11,13–15,62). To investigate the functional consequences of the newly identified RP gene mutations reported here, EBV-immortalized LCLs were generated from peripheral blood of the individuals in this study for ribosome biogenesis analysis. For comparison, two different siRNAs were used to knock down uL6 in HeLa cells (Supplementary Figure S5). As seen on the northern blot of Supplementary Figure S5A, depletion of uL6 leads to a clear accumulation of long early precursors [i.e. 45S, 43S, and 41S, as seen with the (ITS1)-5.8S probe]. Although 32S pre-rRNAs accumulate only slightly, 12S pre-rRNAs are clearly underrepresented (Supplementary Figure S5A). This is evidenced by the 12S/32S ratio which is lower upon depletion of uL6 (Supplementary Figure S5B) and suggests impairment in cleavage at site 4 in the maturation of 28S rRNA (Supplementary Figure S4). A similar defect has been reported in yeast *S. cerevisiae* with an impaired cleavage at site C2 (similar to site 4 in humans) upon uL6 depletion (63). Our data show that the small subunit production is also impacted, with a clear accumulation of 30S and 18S-E pre-rRNAs (5′ITS1 probe, Supplementary Figure S5A). Indeed, defective processing of pre-rRNAs of the large ribosomal subunit often affects 18S rRNA formation too. This is usually due to a delayed/impaired processing at site 2, which then favors direct cleavage at site E (thus leading to an accumulation of 36S and 36S-C pre-rRNAs, as well as 18S-E pre-rRNA; Supplementary Figure S4). This is shown experimentally by a decrease of the 21S/30S ratio and an increase of the 18S-E/21S ratio for cells treated with uL6 siRNAs (Supplementary Figure S5B) and suggests that cleavage of both the 5′ external transcribed spacer (5′ETS) and internal transcribed spacer (ITS1) flanking the 18S rRNA is delayed.

**Figure 2.**
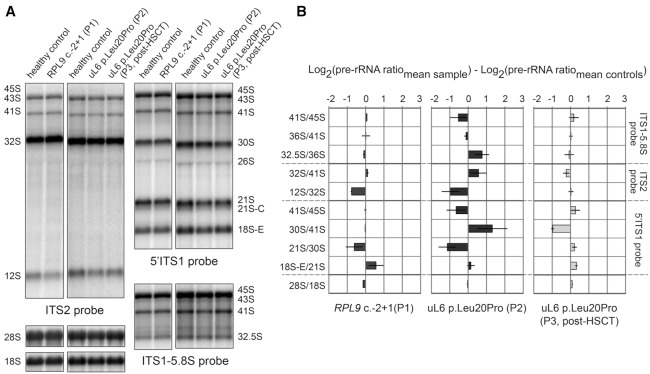
Variants in *RPL9* recapitulate specific pre-rRNA processing defects found in RP depleted cells and show key differences. (**A**) Northern blot analysis of LCLs derived from individuals carrying *RPL9* variants. Radio-labeled probes against ITS2 (left panels), ITS1–5.8S (upper right panels), 5′ITS1 (middle panels), or 18S and 28S (lower right panels) rRNA sequences were used to blot 3 μg total RNA isolated from cells. (**B**) Quantification of three independent experiments analyzing rRNA precursors in LCLs derived from individuals carrying *RPL9* variants using RAMP.

Figure 2 summarizes the northern blot results obtained with LCLs derived from Patients 1 and 2 carrying the variants *RPL9* c.-2+1G>C and uL6 p.Leu20Pro, respectively, and the post-HCT LCLs from P3 (where the uL6 p.Leu20Pro variant is no longer present, see Figure 1B). The pre-rRNA profiles were compared to 2 healthy control LCLs. The phenotypes in cells from Patients 1 and 2 recapitulate key features of the profile observed with *RPL9* siRNAs, namely the decrease of the 12S/32S and 21S/30S ratios (Figure 2A and B). An increase in the 30S/41S ratio (reflecting a decrease in 41S pre-rRNA) was observed only in the uL6 p.Leu20Pro LCLs of P2 and not the others, unveiling some difference in this variant compared to P1 (Figure 2B). The 18S-E/21S ratios were also slightly increased in LCLs from patients, although not as much as in the *RPL9*-depleted HeLa cells. As expected, pre-rRNA from post-HCT LCLs from P3 showed little difference from control cells.

### *RPL9* variants impair ribosomal subunit formation

**Figure 3.**
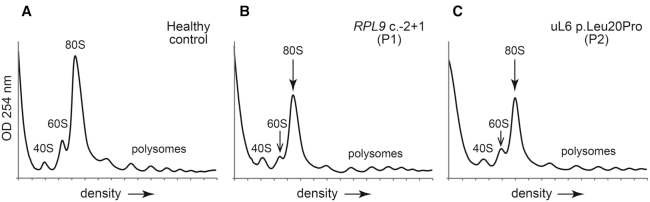
Variants in *RPL9* confer different polysome profile peak ratios. (A–C) Representative polysome profiles of LCLs derived from a healthy control (**A**), a DBA-affected individual (P1) carrying the *RPL9* c.-2+1 variant (**B**), and an individual (P2) carrying the uL6 p.Leu20Pro variant (**C**). or healthy controls. The free 40S and 60S subunits, 80S monosomes, and polysomes are labeled. The reduced 60S peaks in the profiles are indicated with open arrows, the reduced 80S monosomes indicated by filled arrows.

In order to determine how the variants in *RPL9* affect 40S and 60S ribosomal subunit formation, lysates of the HeLa cells transfected with the siRNAs against *RPL9* were first fractionated by ultracentrifugation on sucrose gradients and polysome profiles were analyzed by optical density (Supplementary Figure S5C). These results show that the reduction of *RPL9* leads to a substantial reduction of the 60S large ribosomal subunits, along with a reduction of 80S monosomes, polysomes, and 40S ribosomal subunits peaks that accumulate likely due to the loss of available 60S subunits for joining (Supplementary Figure S5C). Lysates of LCLs from healthy controls or individuals carrying *RPL9* variants were next fractionated on sucrose density gradients and peak sizes measured (Figure 3). A substantial reduction of the 60S large ribosomal subunit, coupled to a reduction of the 80S monosomes, was observed in the polysome profiles of LCLs carrying the *RPL9* c.-2+1 variant (from P1) compared to control cells (Figure 3A and B), consistent with what is observed in Supplementary Figure S5C when uL6 is knocked down in HeLa cells. A similar profile was observed with LCLs carrying the uL6 p.Leu20Pro variant (from P2), but the reduction of the 60S subunit was not as pronounced (Figure 3C). These results indicate that production of the 60S subunit is impaired in P1 and P2 cells, consistent with the pre-rRNA processing defects observed by northern blot in Figure 2.

### Zebrafish models of *RPL9* loss-of-function recapitulate the anemia but not the cancer phenotype

**Figure 4.**
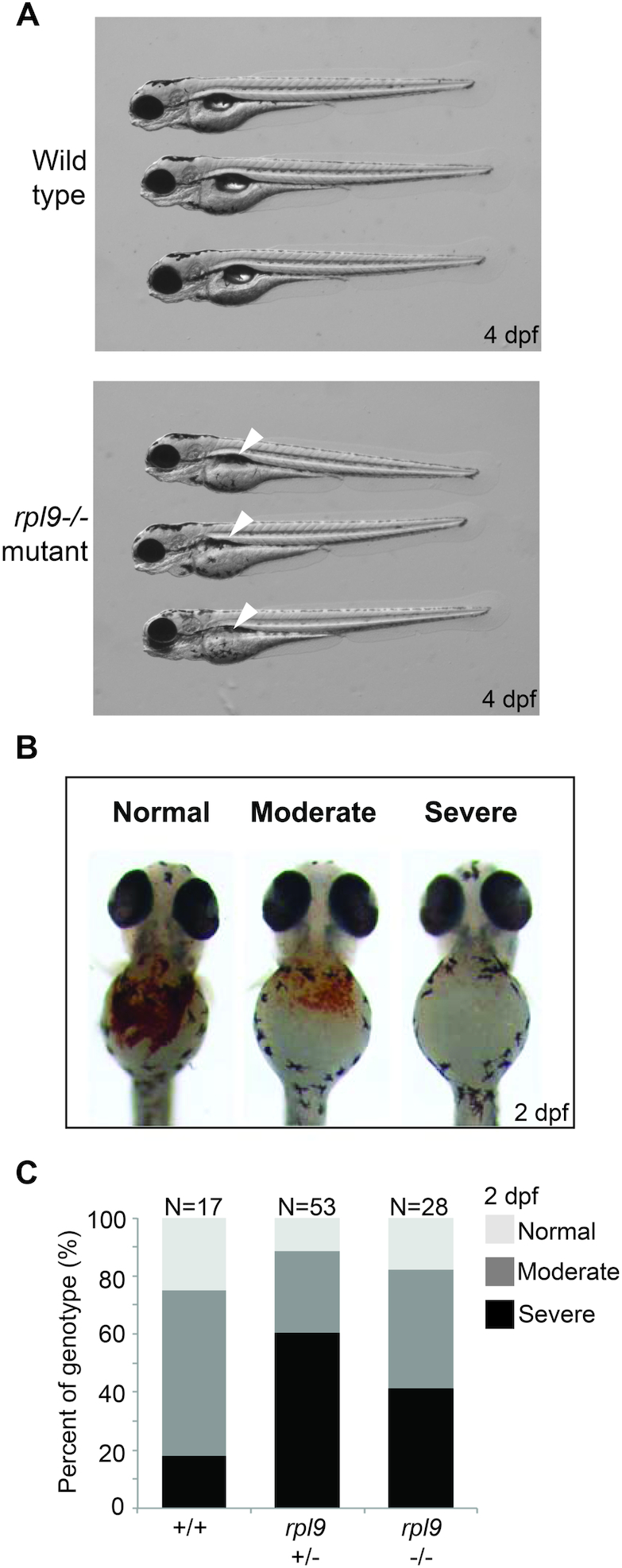
Zebrafish models of *rpl9* loss recapitulate the anemia phenotype of DBA. (**A**) Morphology at 4 dpf of wild type embryos or hi1422 mutants that reduce levels of uL6 protein coded for by *rpl9*. White arrowheads indicate the failure of swim bladder inflation. (**B**) Illustration of scoring embryos at 2 dpf stained with o-dianisidine as having a normal, moderate, or severe phenotype of hemoglobin-expressing cells. (**C**) Scoring and genotypes of o-dianisidine stained embryos from clutches of hi1422 matings stained at 2 dpf.

Many (if not most) homozygous mutant zebrafish models of RP gene knockout by viral insertion (64) reveal a depletion of hemoglobin-expressing cells when embryos are stained with o-dianisidine (65,66). Interestingly, several of these zebrafish lines (17/28) as heterozygous adults are also prone to developing peripheral nerve sheath tumors (MPNSTs) (46). We characterized the hematopoietic phenotype of a zebrafish line carrying a viral insertion in the *rpl9* gene that was previously generated (64). Figure 4A shows that at 4 days post-fertilization (dpf) embryos homozygous for the viral insertion in *rpl9* (*rpl9^−^^/^^−^*) fail to inflate their swim bladders and subsequently die by 6–7 dpf. Staining of wild type and mutant zebrafish embryos at 2 dpf with *o*-dianisidine (followed by genotyping of single embryos) reveals different severities of the loss of hemoglobin-expressing cells, illustrated in Figure 4B. In clutches of embryos generated from mating two heterozygous *rpl9* mutant fish we found that both hetero- and homozygous embryos fail to generate hemoglobin-expressing cells to the same degree as wild type fish, in effect phenocopying the DBA anemia phenotype (Figure 4C). However, the *rpl9* heterozygous line is reported to be a non-tumor prone line, suggesting that at least in zebrafish models the mere reduction of *rpl9* mRNA is insufficient to drive cancer (67).

### Erythroid cell culture assays reveal primary CD34^+^ cells carrying *RPL9* 5′UTR variants fail to proliferate

**Figure 5.**
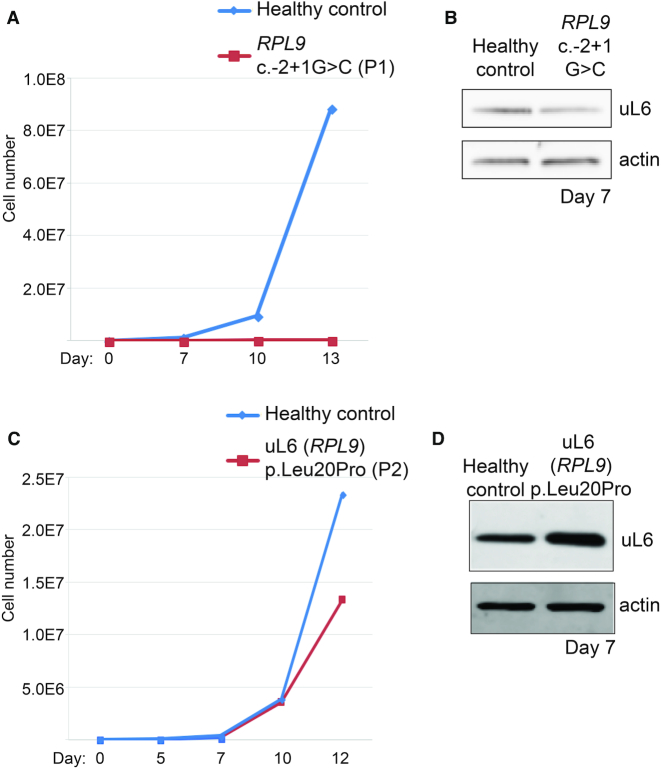
Erythroid cell culture assays of primary CD34^+^ cells reveal proliferation defects only in cells with 5′UTR variants. (**A**) Growth curves of CD34^+^ cells isolated from peripheral blood of the DBA-affected individual carrying the *RPL9* c.-2+1 variant (P1, red) compared to erythroid cells from a healthy control (blue). **(B)** Western blotting of lysates from cells in (A) collected at Day 7 and probed with antibodies against uL6 protein. (**C**) Growth curves of CD34^+^ cells isolated from the individual carrying the uL6 (*RPL9*) p.Leu20Pro variant (P2, red) compared to cells from a healthy control (blue). (**D**) Western blotting of lysates from cells in (C) collected at Day 7 and probed with antibodies against uL6 protein.

Erythroid cell culture assays show that hematopoietic progenitor cells can reveal reduced proliferation rates, delayed differentiation, and increased TP53-induced apoptosis in a manner that is largely dependent on which RP gene is mutated or knocked down (25). To investigate the effects of the different *RPL9* variants, CD34^+^ primitive erythroid progenitor (BFU-e) cells from Patients 1 and 2 were isolated from peripheral blood and plated in erythroid cell culture medium, then allowed to grow for 12–15 days alongside CD34^+^ BFU-e cells isolated from a healthy individual. Figure 5A shows that CD34^+^ cells derived from P1, the DBA-affected individual carrying the *RPL9* c.-2+1G>C variant in the 5′UTR, do not proliferate in red cell culture medium compared to cells from a healthy control. FACS analysis of cells from P1 compared to a healthy control also reveals higher levels of annexin V on days 7 and 13 and higher expression levels of CD34 at day 7, suggesting higher levels of apoptosis and delayed differentiation, respectively, in the cells from P1 (Supplementary Figure S6A–E). In line with the higher levels of annexin V suggesting increased apoptosis, western blots in Supplementary Figure S6F from cells collected at Day 7 reveal increased levels of TP53 and its downstream target CDKN1A (p21). Figure 5B reveals that the expression of uL6 on Day 7 is lower in cells carrying the 5′UTR variants. In contrast, Figure 5C reveals that CD34^+^ cells derived from P2 carrying the p.Leu20Pro missense variant in uL6 (*RPL9*) proliferate normally compared to cells from a healthy control. Consistent with the fact that the P2 has no medical history of anemia, FACS analysis of P2 cells do not reveal any notable differences in expression of the erythrocyte differentiation markers Band3 or glycophorin A (GPA) compared to cells from a healthy control (Supplementary Figure S7A and B). Moreover, in contrast to cells carrying the 5′UTR variant in Figure 5B, western blots in Figure 5D show that cells carrying the p.Leu20Pro variant do not reveal any reduction of uL6 protein. This result is consistent with the polysome profiles in Figure 3 revealing a greater reduction of 60S ribosomal subunits in the cells carrying the 5′UTR variant compared to the p.Leu20Pro variant.

### The 5′UTR variant of *RPL9* is linked to TP53 stabilization in LCLs

**Figure 6.**
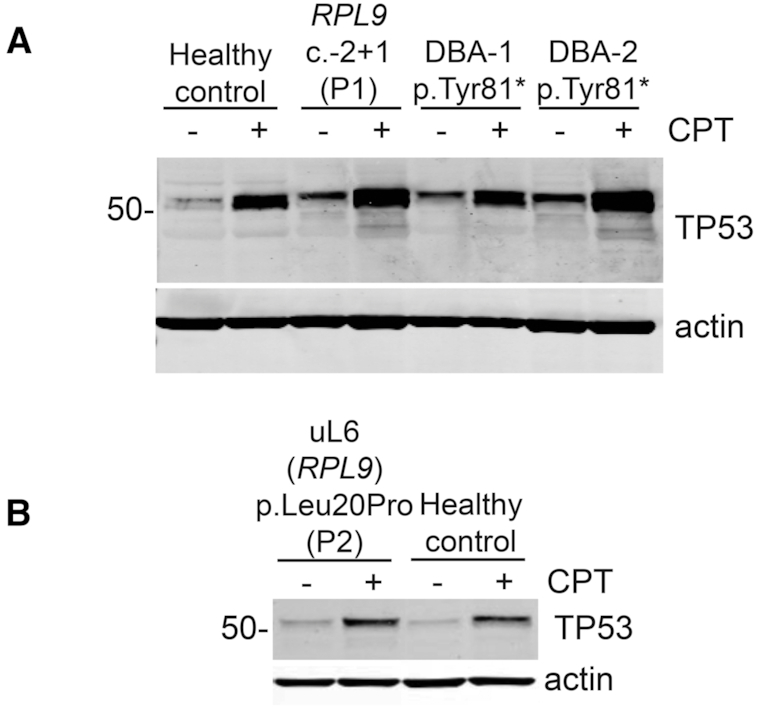
TP53 protein is stabilized in cells carrying the *RPL9* 5′UTR variant. (**A**) Western blots of LCL lysates either treated for 6 h with a DMSO vehicle control (−) or 100 nM camptothecin (CPT, +) probed with antibodies against TP53. LCLs are derived from a healthy control, the DBA-affected individual carrying the *RPL9* c.-2+1 variant (P2), or two unrelated DBA-affected individuals carrying truncations in *RPL15* (21). (**B**) Similar western blots as in (A) using LCLs derived from the individual carrying the uL6 (*RPL9*) p.Leu20Pro variant (P3).

One of the potential mechanisms behind the inability of CD34^+^ cells to properly proliferate in DBA-affected individuals is that the RP variant drives ribosomal stress which in turn activates the TP53 pathway resulting in TP53 stabilization and initiation of apoptosis (68). This TP53 stabilization is shown in the differentiating erythroid cells carrying the 5′UTR variant in Supplementary Figure S6F. To confirm this result, Figure 6 shows western blotting of LCLs derived from a healthy control, the DBA-affected individual carrying the 5′UTR variant in *RPL9* (P1, c.-2+1G>C), two other DBA-affected individuals (carrying truncating variants in *RPL15* (21)), and from P2 carrying the uL6 (*RPL9*) p.Leu20Pro missense variant. LCLs were treated with DMSO (as a vehicle control) or the topoisomerase inhibitor camptothecin (CPT) as a positive control for 6 h before lysis. The blots were then probed with antibodies against TP53. Figure 6A and B show that LCLs derived from a healthy control reveal minimal expression of TP53 under basal (DMSO-treated) conditions, and the expected stabilization of TP53 upon a 6-hour treatment with CPT. In contrast, Figure 6A shows that LCLs derived from all the DBA-affected individuals including P1 with the *RPL9* c.-2+1 variant reveal substantially more stabilization of TP53 at basal levels, suggesting activation of the TP53 pathway even in the absence of exogenous stress. The addition of CPT to these cells results in further stabilization of TP53. In contrast to the LCLs derived from DBA-affected individuals, Figure 6B shows that cells carrying the uL6 (*RPL9*) p.Leu20Pro missense variant do not reveal any basal level stabilization of TP53. These results are in line with the results in Figure 5 and Supplementary Figure S6 suggesting that cells carrying the p.Leu20Pro missense variant proliferate normally and are not subject to apoptosis as observed in the cells from the DBA-affected individual.

### The uL6 p.Leu20Pro variant is linked to impaired translational fidelity

**Figure 7.**
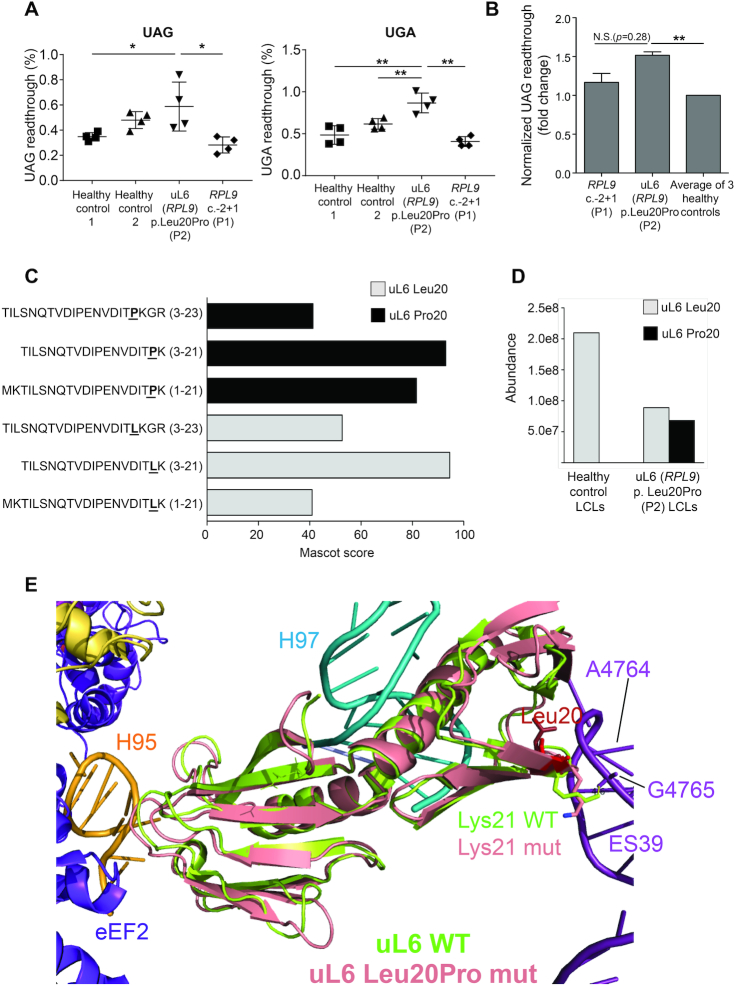
Cells carrying the uL6 p.Leu20Pro variant reveal translational fidelity defects. (**A**) Cell-based assay to measure UAG and UGA stop codon readthough using bicistronic luciferase reporters transfected in LCLs derived from individuals carrying the *RPL9* c.-2+1G>C variant (P2), the uL6 p.Leu20Pro variant (P3), or unrelated healthy controls. *P < 0.05, **P < 0.01. (**B**) Cell-free assay measuring the level of UAG stop codon readthrough in ribosomes purified from LCLs carrying the *RPL9* c.-2+1G>C (P2) or uL6 p.Leu20Pro (P3) variant compared to LCLs from three unrelated healthy controls. Experiments were performed three times in duplicate with two different preparations of ribosomes per cell type; error bars represent standard error. To determine statistical significance, a paired Student's t-test was applied. **P < 0.01. (**C**) MS-based proteomic characterization of ribosomes purified from LCLs of P2 carrying the uL6 p.Leu20Pro variant (*N* = 3). Sequences and scores of peptides identified by Mascot search engine and covering the position 20 of uL6 are presented. On the *y*-axis the amino acid in position 20 of uL6 is in bold font and underlined. (**D**) Quantification of the abundance of uL6 peptides carrying Pro20 or Leu20 measured in purified ribosomes from LCLs generated from a healthy control or P2. (**E**) Predicted structure of uL6 p.Leu20Pro variant protein (in pink) superimposed on the known structure of wild type uL6 (in lime green). Helix 95 of the SRL is shown in gold, amino acid 20 (aa20) is shown in red, rRNA helix 97 is shown in cyan, rRNA extension segment 39 (ES39) is shown in light purple, eEF2 is shown in dark purple, and the positions of the Lys21 (wild type in lime green, variant in pink) are indicated with arrows.

It has been previously reported that the somatic *RPL10* gene variant (uL16 p.Arg98Ser) linked to T-ALL in humans drives defects in translational fidelity by increasing frequencies of programmed −1 ribosomal frameshifting (-1 PRF) in yeast models (36). In order to determine if the uL6 (*RPL9*) p.Leu20Pro variant that is linked to cancer also reveals translational fidelity defects, we measured frameshifting and stop codon readthrough in two different assays with dual luciferase reporter vectors. First, we transfected deidentified LCLs [from a healthy control, P1 carrying the c.-2+1G>C variant or P2 carrying the uL6 (*RPL9*) p.Leu20Pro variant] by nucleofection with reporters designed to measure −1 or +1 PRF or readthrough of stop codons UAA, that have been previously described (28). Supplementary Figure S8 shows that none of the LCLs tested in this assay reveal defects in −1 or +1 PRF or in readthrough of the UAA stop codon. However, Figure 7A shows that LCLs carrying the uL6 (*RPL9*) p.Leu20Pro variant do reveal significant increases (*P* < 0.05 and 0.01) in the readthrough of UAG and UGA stop codons, with the most significant increase being a 2-fold increase of UGA readthrough compared to healthy control LCLs and those carrying the 5′UTR variant. This observation was supported by a separate cell-free assay in Figure 7B designed to measure the translational fidelity of purified ribosomes isolated from the LCLs that has also been previously described (55). This assay showed that only ribosomes isolated from LCLs carrying the uL6 (*RPL9*) p.Leu20Pro variant (but not the *RPL9* c.-2+1G>C variant) revealed a significant increase in the readthrough of the UAG stop codon over ribosomes purified from healthy control LCLs (Figure 7B).

In order to validate that the ribosomes from the LCLs eliciting the translation fidelity defect do in fact carry the uL6 p.Leu20Pro variant protein, we performed a MS-based proteomic analysis on triplicate purifications of mature ribosomes from P2 LCLs. This allowed the reliable identification of six different peptides mapping to the N-terminal of uL6. Three of these peptides carry a leucine residue at amino acid position 20 and correspond to the wild type sequence of *RPL9*, whereas the three others carry a proline residue at this position and correspond to the *RPL9* variant sequence (Figure 7C), indicating that both versions are present in mature ribosomes of P2. Since the identified Leu20 and Pro20 containing peptides are very close in sequence, we used label-free quantification to estimate and compare the abundances of the two uL6 peptide versions in ribosomes purified from P2 LCLs. As shown in Figure 7D, it revealed that these ribosomes from P2 LCLs contain about the same quantity of uL6 p.Leu20 and of uL6 p.Pro20 peptides compared to ribosomes purified from healthy control LCLs that exclusively contain uL6 p.Leu20 peptides. These data suggest that the uL6 proteins generated from the wild type and mutated alleles of *RPL9* contribute about equally to the structure of mature ribosomes in LCLs carrying the uL6 p.Leu20Pro variant.

In order to visualize how the incorporated uL6 p.Leu20Pro protein might alter the ribosome we used Pymol software to model the uL6 structure and its surroundings with either the WT or variant version of the amino acid residue 20. Structural visualization of uL6 is shown in Figure 7E, where the protein is found situated in the ribosome between rRNA helix H97 (in cyan) and expansion segment 39 (ES39, in light purple). The WT version containing Leu20 is shown (in lime green), superimposed with the predicted structure of the variant uL6 p.Leu20Pro protein (in pink). The position of amino acid 20 is shown in red. This model predicts that a p.Leu20Pro variant changes the orientation of the end of a beta sheet, which in turn alters the angle of Lys21. Normally, Lys21 interacts with the phosphate group between A4764 and G4765 located in ES39 of the 28S rRNA (measured distance is 4.6 Angstroms, suggesting that this is a salt bridge) (59). A Pro20 is predicted to alter the orientation of Lys21, disrupting this interaction. This in turn is predicted to weaken the interaction between uL6 and the 28S rRNA. The model also predicts that the p.Leu20Pro variant has some large-scale effects on uL6 folding. These are predicted to affect the other side of the protein, particularly the loop domain that interacts with Helix 95 (H95) of the Sarcin/ricin loop (SRL, shown in gold). Importantly, the SRL plays a key role in activation of the GTPase activity of both the elongation factors (eEF1A and eEF2) as well as the release factor eRF1 (eEF2 is shown in dark purple). Specifically, the model predicts that this loop is displaced toward the SRL, creating the potential for a steric clash. We suggest that this may displace the SRL, interfering with its ability to optimally interact with the elongation and release factors. Consistent with the translational fidelity assays, it also suggests that this potentially altered interaction affects ribosome function, i.e. the ability to faithfully decode mRNAs.

### *RPL9* variants drive different metabolic profiles in LCLs

**Figure 8.**
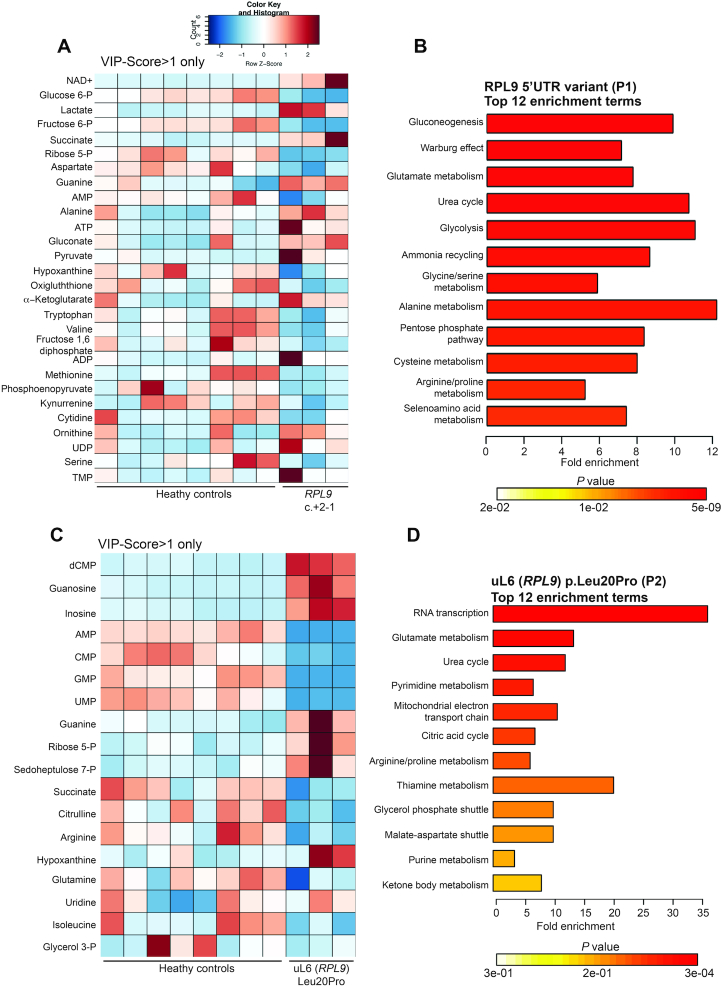
Metabolic profiles and enrichment analysis of LCLs carrying *RPL9* variants. (**A**) Metabolic profile heat map showing *Z*-scores of LCLs derived from three unrelated healthy controls compared to cells from DBA-affected individual (P1) carrying variants in the 5′UTR of *RPL9*. (**B**) Enrichment analysis (using Metabolanalyst 3.0 online software) of significantly changed metabolites from (A). (**C**) Metabolic profile heat map showing *Z*-scores of LCLs derived from three unrelated healthy controls compared to cells from an individual carrying the uL6 (*RPL9*) p.Leu20Pro variant (P2). (**D**) Enrichment analysis of significantly changed metabolites from (B). For (A) and (C) only metabolites with comparative VIP-Scores >1 (out of 63 metabolites measured) are listed.

In order to determine how the different *RPL9* variants affect cellular metabolism, we used a MS-metabolomics approach to measure the levels of over 120 polar metabolites in LCLs derived from affected individuals and compared them to cells from three unrelated healthy controls. Figure 8A shows a heat map of metabolites in LCLs carrying the 5′UTR variant compared to the healthy controls sorted according to their Variable Importance in Projection (VIP) score, with only metabolites with a VIP score >1 shown. The enrichment analysis performed with Metabolanalyst 3.0 (69) showing the top 12 terms is shown for this profile in Figure 8B. These results suggest that glycolysis in the cells with the 5′UTR variant in *RPL9* is impaired and that the cells have switched to gluconeogenesis, the pathway that results in the formation of glucose from non-carbohydrate carbon sources such as gluconogenic amino acids. The catabolism of amino acids produces ammonia (70). As such, these cells also reveal significant enrichment of the terms ‘ammonia recycling’ and the ‘urea cycle’, presumably reflecting an excretion of the ammonia (which is highly toxic) resulting from the upregulated catabolism of amino acids during gluconeogenesis.

A remarkably different metabolic profile and set of enrichment terms are revealed when the same healthy control LCLs are compared to cells carrying the missense uL6 (*RPL9*) p.Leu20Pro variant. Figure 8C shows the most significantly downregulated metabolites are nucleotides AMP, CMP, GMP and UMP while the most significantly upregulated metabolites are dCMP, guanosine, and inosine. In contrast to the LCLs carrying the 5′UTR variant, only the amino acid arginine is found significantly downregulated in LCLs carrying the missense variant. Figure 8D shows that the top enrichment term in the profiles from LCLs carrying the missense variant is ‘RNA transcription’ with a 35-fold enrichment (due to the significant reduction of nucleotides) and there is no indication of impaired glycolysis as in the cells with 5′UTR variant. These results suggest that there are some substantial and fundamental differences in the metabolic consequences of the two variants.

## DISCUSSION

This study reports that different variants of *RPL9*, a gene that has not been definitively associated with human disease before, are linked to DBA or multiple cancer incidences. Exome sequencing of trios revealed an individual with DBA (P1) carrying a variant of *RPL9* in the 5′UTR. Exome sequencing of tumor and germ line DNA from a family with multiple cancer incidences (P2 and P3) brought to light a missense p.Leu20Pro variant in uL6 (*RPL9*) that is predicted to be damaging. All the variants tested showed defects in the processing of pre-rRNA and reductions of 80S monosomes that mimic the knockdown of *RPL9* in HeLa cells (Figures 2, 3, and Supplementary Figure S5), which strongly suggests that the variants are pathogenic.

Remarkably, the downstream effects of these seemingly similar pre-rRNA defects appear to be largely dependent on the type of variant. The stabilization of TP53 is only observed in cells carrying the *RPL9* 5′UTR variant (Figure 6 and Supplementary Figure S6F). In turn, only erythroid cells cultured from primary CD34^+^ cells carrying the 5′UTR variant have defects in proliferation and differentiation and undergo apoptosis (Figure 5). In contrast, the p.Leu20Pro missense variant has little effect on CD34^+^ cells in the erythroid cell culture assay (Figure 5). This is well in line with the fact that the individuals carrying the missense variant do not have any bone marrow failure or anemia phenotype.

The polysome profile results in Figure 3 indicate that the cells carrying the 5′UTR variant of *RPL9* suffer a very substantial reduction of the number of 60S ribosomal subunits. Given that this variant does not modify the uL6 protein itself, it is likely that it impairs translation of *RPL9* mRNA, which results in haploinsufficiency of this ribosomal protein gene as classically described in DBA. Consistently, TP53 is activated, presumably in response to the ribosomal stress resulting from uL6 limiting amount. The p.Leu20Pro variant in turn shows much less impairment of 60S subunit production on the gradients. While pre-rRNA processing is affected in this patient, this variant does incorporate in the mature ribosome and impairs its translational fidelity by increasing the readthrough of UAG and UGA stop codons in a way that is not observed with the 5′UTR variant (Figure 7). As such, the uL6 p.Leu20Pro variant may be described as a gain-of-function, since it is changing how certain mRNA stop codons are interpreted. The structural analysis in Figure 7E suggests that the incorporation of the uL6 p.Leu20Pro variant may affect ribosomal domains such as the peptidyltransferase center and/or the Sarcin/Ricin loop (SRL) that are critical for maintaining translational fidelity of the ribosome (71). However, bona fide structural analysis of 60S ribosomal subunits incorporating the uL6 p.Leu20Pro variant will be required to make any conclusive statements in this regard.

Some of the most striking differences of the downstream effects of these *RPL9* variants may be found in the metabolic profiles. The profiles and enrichment analysis of LCLs derived from the DBA-affected individual (Figure 8A and B) suggest that the 5′UTR variant impairs glycolysis and causes the cells to switch to gluconeogenesis as a means of generating glucose from non-carbohydrate sources, such as glucogenic amino acids. This catabolism of amino acids likely results in an increase of highly toxic ammonia which the cells then excrete by upregulating the urea cycle (another highly enriched term in the analysis of Figure 8B). In contrast, cells carrying the missense variant reveal a dramatic reduction of nucleotides that is not observed in the cells with the 5′UTR variant (Figure 8C). This is coupled to changes in glutamate metabolism that suggest an accelerated synthesis of RNA, a term that dominates the enrichment analysis in Figure 8D. A likely explanation for this profile and accompanying enrichment analysis is that the cells carrying the missense variant are signaling to increase the manufacturing of new ribosomes, which by definition would require a strong upregulation of rRNA synthesis and a depletion of nucleotide pools. Such a metabolic change is not observed with the 5′UTR variant, which may be partly explained by previous observations showing that cells carrying DBA-related RP gene mutations slow down the overall rate of protein synthesis compared to cells carrying RP gene mutations that predominantly affect translational fidelity (28). The switch to gluconeogenesis observed in the cells carrying the 5′UTR variant also suggests that amino acids in these cells are being preferentially used to supply the cell with energy and not for protein synthesis. The stabilization of TP53 in these cells that we observe may be an important factor governing this metabolic switch, as it is known that TP53 is able to suppress glucose transport into cells in addition to inducing gluconeogenesis (72).

We propose a genotype:phenotype model for *RPL9* whereby variants that drive the reduction of uL6 are sufficient to drive DBA but not cancer, in contrast to variants that impair translational fidelity. This is supported by the fact that heterozygous *rpl9* zebrafish are not reported to develop tumors (67), although our results in Figure 4 suggest that both the hetero- and homozygous mutant embryos recapitulate the DBA anemia phenotype. We recognize that in the absence of a mouse model it is difficult for us to state definitively that the cancers found in individuals P2 and P3 are directly linked to the *RPL9* p.Leu20Pro variant. The evidence suggesting the connection is as follows: (i) There are no other obvious driver mutations found in the exomes of either tumor subject to sequence analysis. (ii) The p.Arg98Ser variant in *RPL10*/uL16, linked to human T-ALL, drives translational fidelity defects including stop codon readthrough in yeast (36). (iii) An increasing body of evidence is linking RP gene variants to different forms of human cancer. This includes DBA-linked and other inherited RP variants that increase the predisposition to cancer as well as variants that arise spontaneously (4,35,73). (iv) The osteosarcoma and AML described here are cancers frequently observed in DBA patients. So, although we must remain cautious when describing the potential impact of the uL6 p.Leu20Pro variant, it does seem possible that the translational fidelity defects are contributing to instability in cells that ushers them closer to the tipping point of malignancy. Supporting this mechanism is the finding that yeast cells with a defective non-stop decay pathway (and synthesizing aberrant proteins coded from 3′UTRs) reveal an increased sensitivity to oxidative stress, which has well-known links to cancer (74,75). This will be a very interesting area of future study as more variants in human genes coding for components that regulate mRNA translation are identified and characterized.

In sum, this study reports different variants of *RPL9*, a gene that has not been previously associated with human disease, drive similar defects in pre-rRNA processing yet have remarkably different impacts downstream of this initial ribosome biogenesis defect. The results suggest a model whereby cells carrying a DBA-linked RP variant generate fewer 60S ribosomal subunits overall, reduce glycolysis, switch to gluconeogenesis, and catabolize amino acids as a carbon source. This is accompanied by reduced cell proliferation, increased TP53 stabilization, and apoptosis. In contrast, the incorporation of the uL6 p.Leu20Pro variant into 60S ribosomal subunits does not severely affect the production rate of 60S subunit, but compromises the fidelity of the ribosome by increasing the readthrough of stop codons. Taken together, these results underscore the widening landscape of cellular and clinical phenotypes that are associated with human RP gene variants. They also suggest that despite similar pre-rRNA processing defects in cells carrying different variants of the same RP gene, the downstream effects can diverge dramatically and in ways that may help to clarify the biology underlying these complex genotype:phenotype relationships.

## Supplementary Material

gkz1042_Supplemental_File

## Notes

Present address: Evelien Zonneveld-Huijssoon, University Medical Center Groningen, 9713 GZ Groningen, The Netherlands.

Present address: Alyson W. MacInnes, Philips Research, Department of Precision Diagnostics, 5656 AE Eindhoven, The Netherlands.
