# Supplementary material for: Ribosomal protein gene RPL9 variants can differentially impair ribosome function and cellular metabolism
Source: Nucleic Acids Res. 2019 Dec 4;48(2):770–87. doi: 10.1093/nar/gkz1042 (PMC6954397; doi:10.1093/nar/gkz1042)
Supplement: gkz1042_Supplemental_File [file gkz1042_supplemental_file.pdf]

Tumor 1													
Chromosome	Start position	End position	Reference	Variant	Reads	Variation reads	Gene name	Reference Amino Acid	Mutation Amino Acid	Synonymous	Amino Acid position	mRNA changes
chr7	101265374	101265374	A	T	95	21	MYL10			FALSE	-1	
chr19	54444763	54444763	G	A	49	11	CACNG7	S	N	FALSE	155	464G>A

Tumor 2												
Chromosome	Start position	End position	Reference	Variant	Reads	Variation reads	Gene name	Reference Amino Acid	Mutation Amino Acid	Synonymous	Amino Acid position	mRNA changes
chr1	93202030	93202030	G	T	85	28	EVI5	A	D	FALSE	69	206C>A
chr5	132652300	132652300	G	A	93	35	FSTL4	L	F	FALSE	152	454C>T
chr7	128517835	128517835	C	T	32	16	KCP			FALSE	-1	
chr11	69063542	69063542	G	A	123	45	MYEOV	G	R	FALSE	209	625G>A
chr14	20345206	20345206	G	T	301	61	OR4K2	W	C	FALSE	260	780G>T
chr19	52272633	52272633	G	A	112	40	FPR2	R	Q	FALSE	241	722G>A
chr22	42053003	42053003	A	G	37	18	XRCC6	K	R	FALSE	463	1388A>G
chr22	51010473	51010473	C	T	46	19	CPT1B	A	T	FALSE	513	1537G>A

Table S1. Exome sequencing reveals variants in tumors from P3. Tumor 1 represents the exome sequencing results from the osteosarcoma, Tumor 2 represents results from the AML. Not shown is the *RPL9* variant, which was found in both tumor types.

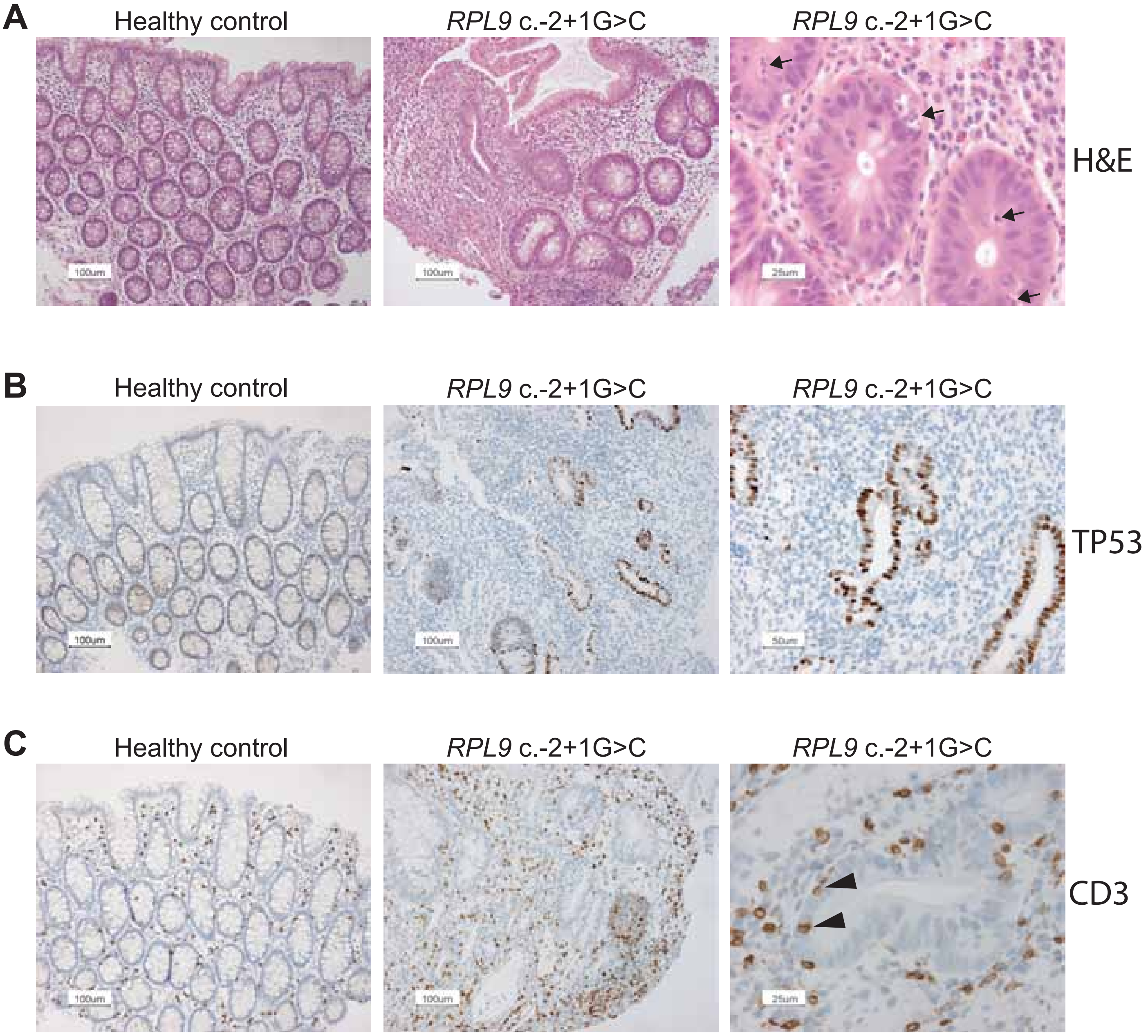

Figure S1 (related to Figure 1 and Table 1). Colon tissue biospy revealing TP53 stabilization in a DBA-affected individual with colitis carrying the *RPL9* c.-2+1G>C variant. A) Haematoxylin and eosin staining of biopsied colon tissue taken from a DBA-affected individual or a healthy age-matched control. Arrows point to nuclear fragmentation typical of apoptotic bodies.
B) Immunohistochemistry of biopsied colon tissue stained with antibody against TP53 (healthy control left panel, DBA-affected individual with the *RPL9* variant center and left panels). Note intense TP53 staining in the epithelial cells in basal half of the crypts. **C)** Imunohistochemistry of biopsied colon tissue stained with antibodies against CD3 (healthy control left panel, DBA-affected individual with the *RPL9* variant center and left panels). Arrowheads indicate CD3+ cells infiltrating the epithelium.

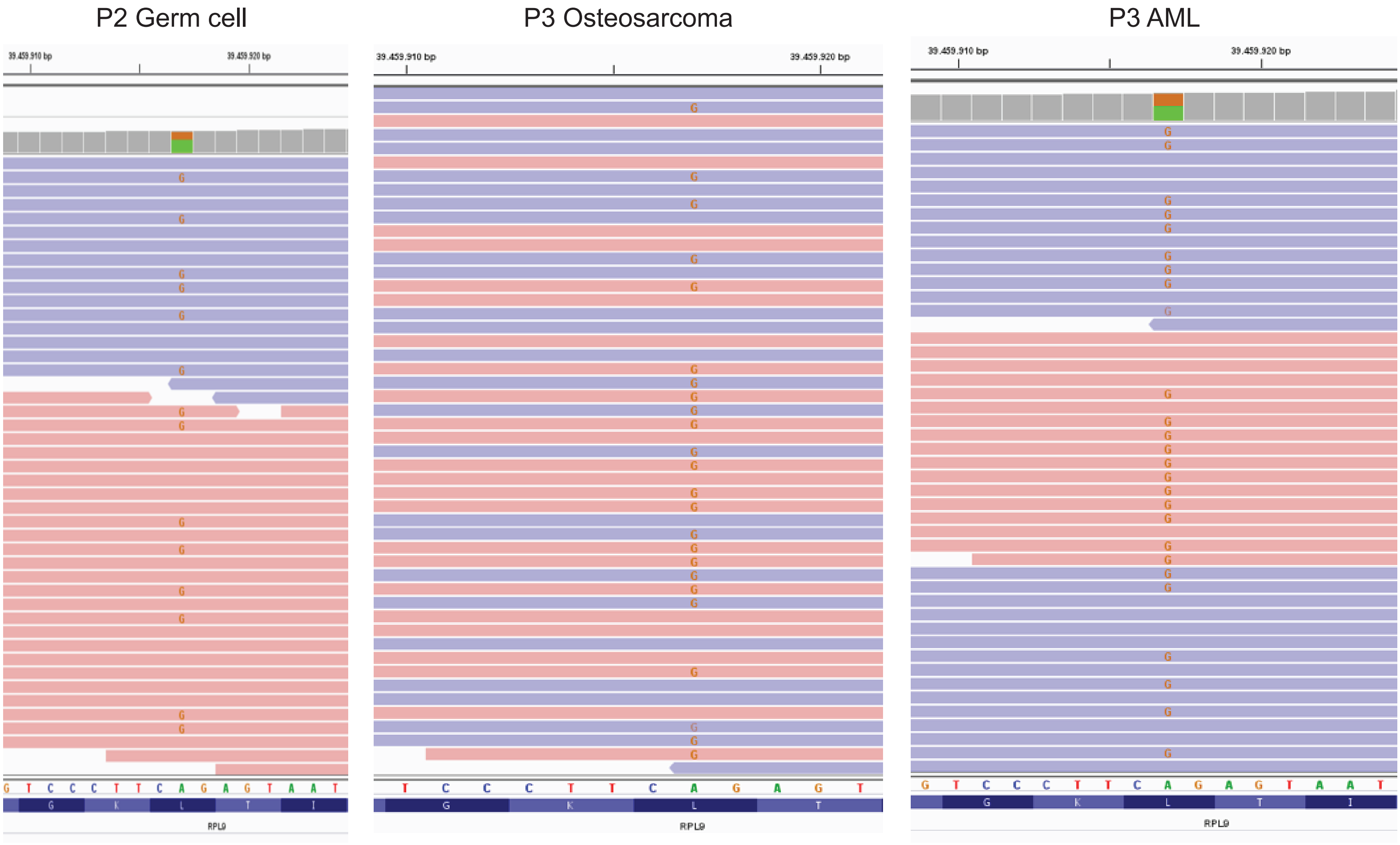

Figure S2. *RPL9* varaints are found in individuals P2 and P3. Integrated genomics viewer (IGV) screen shots show the exome sequencing results of the different tissues from P2 and P3. From top to bottom P2 germ cell, P3 osteosarcoma, and P3 AML.

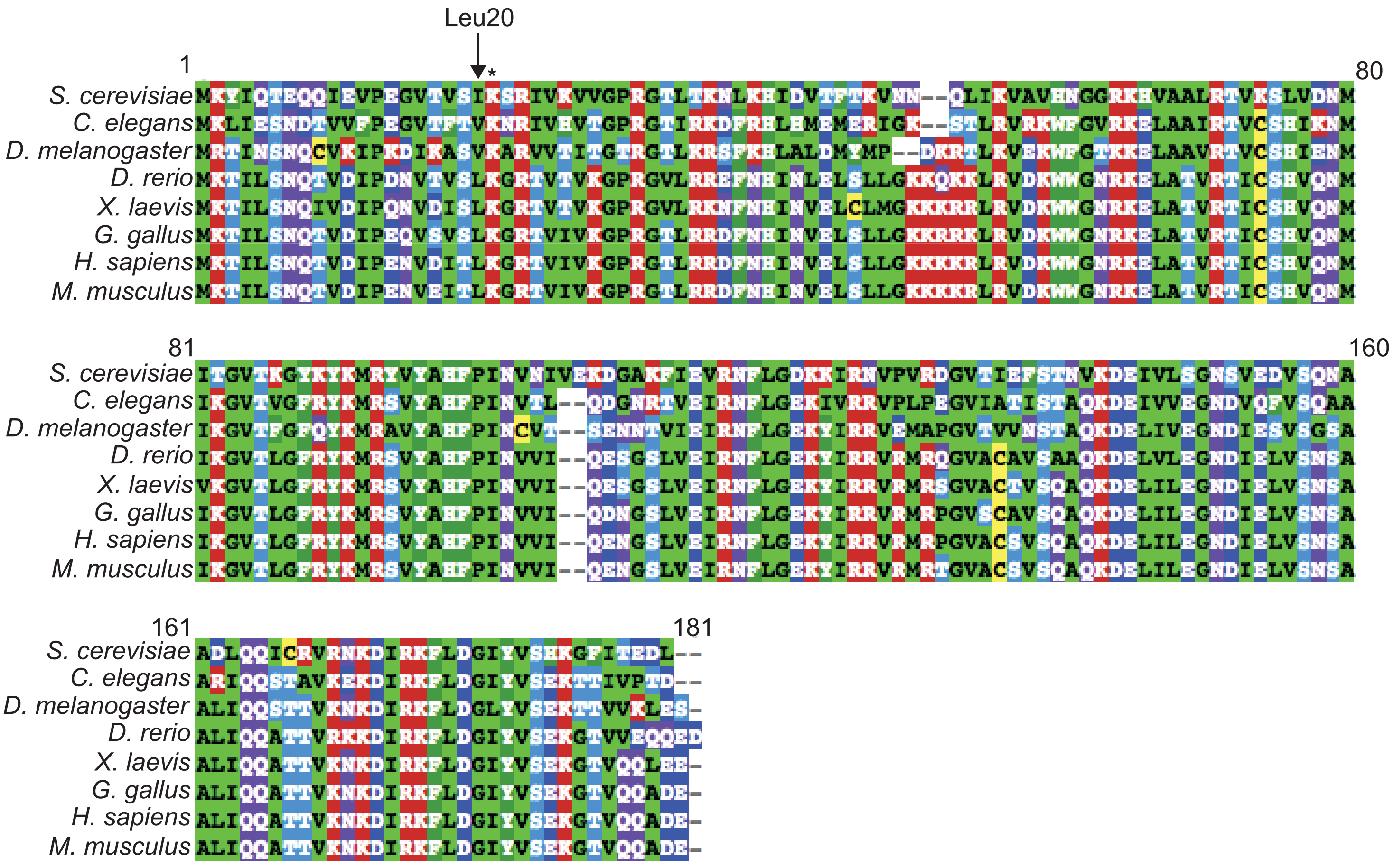

Figure S3 (related to Figure 1). Multiple sequence alignment (MSA) of uL6 proteins. The human Leu20 residue in uL6 (*RPL9*) precedes a universally conserved Lys21 residue (indicated with *) and is itself conserved in mammals, birds, frogs, and fish. The hydrophobicity of residue 20 is also conserved in eukaryotes, as yeast, worms, and flies reveal either Ile20 or Val20 in this position. Color coding is based on the physicochemical properties of the amino acids,with hydrophobic amino acids shown in green (black font), large hydrophobic in green (white font), negatively charged in dark blue, positively charged in red, cysteines in yellow, polar in purple, and small alcohol in light blue.

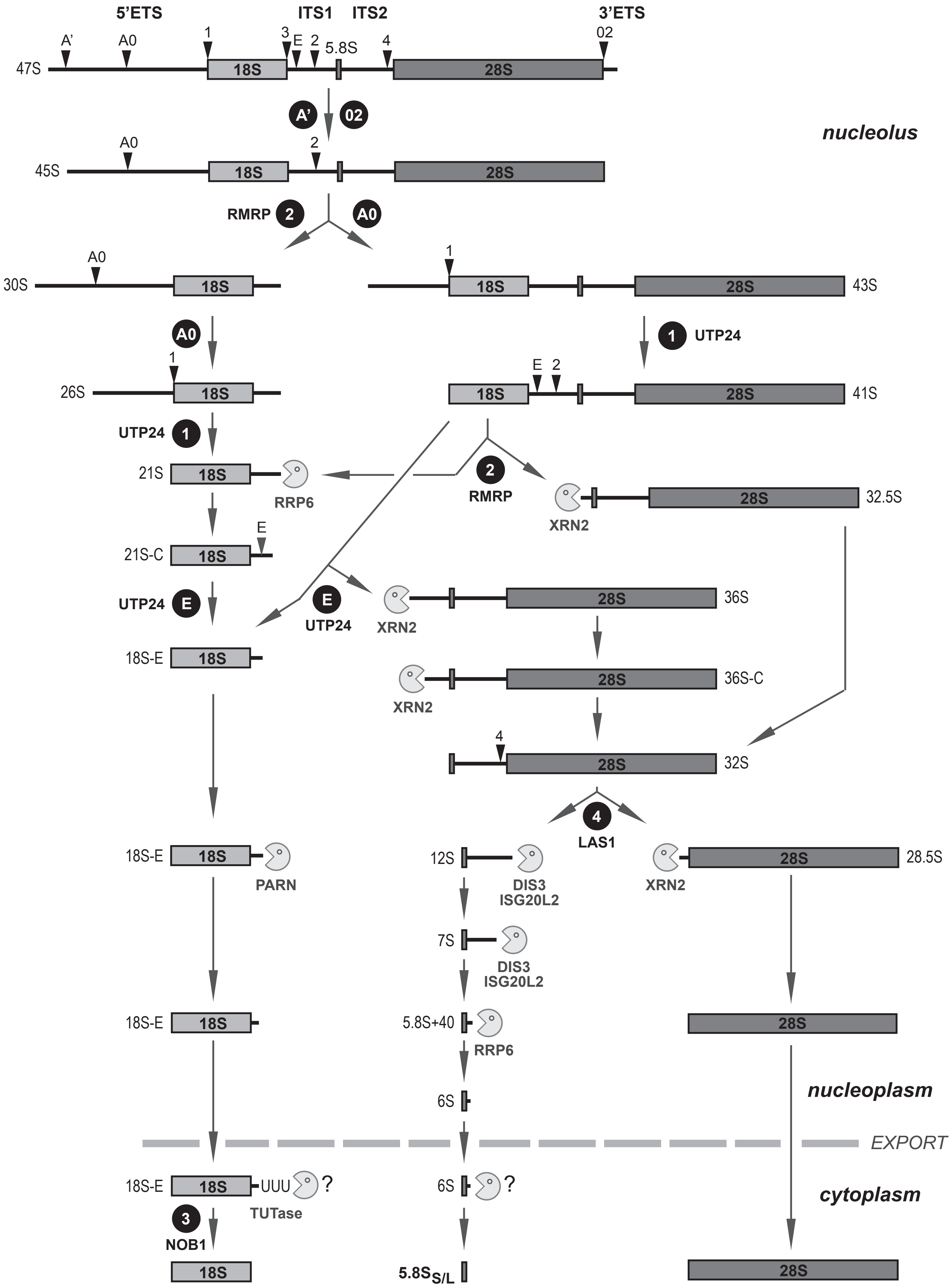

Figure S4 (related to Figure 2). A schematic of pre-rRNA processing in human cells. Major endonucleolytic cleavage steps are labeled with black circles, the relevent enzymes are also noted.

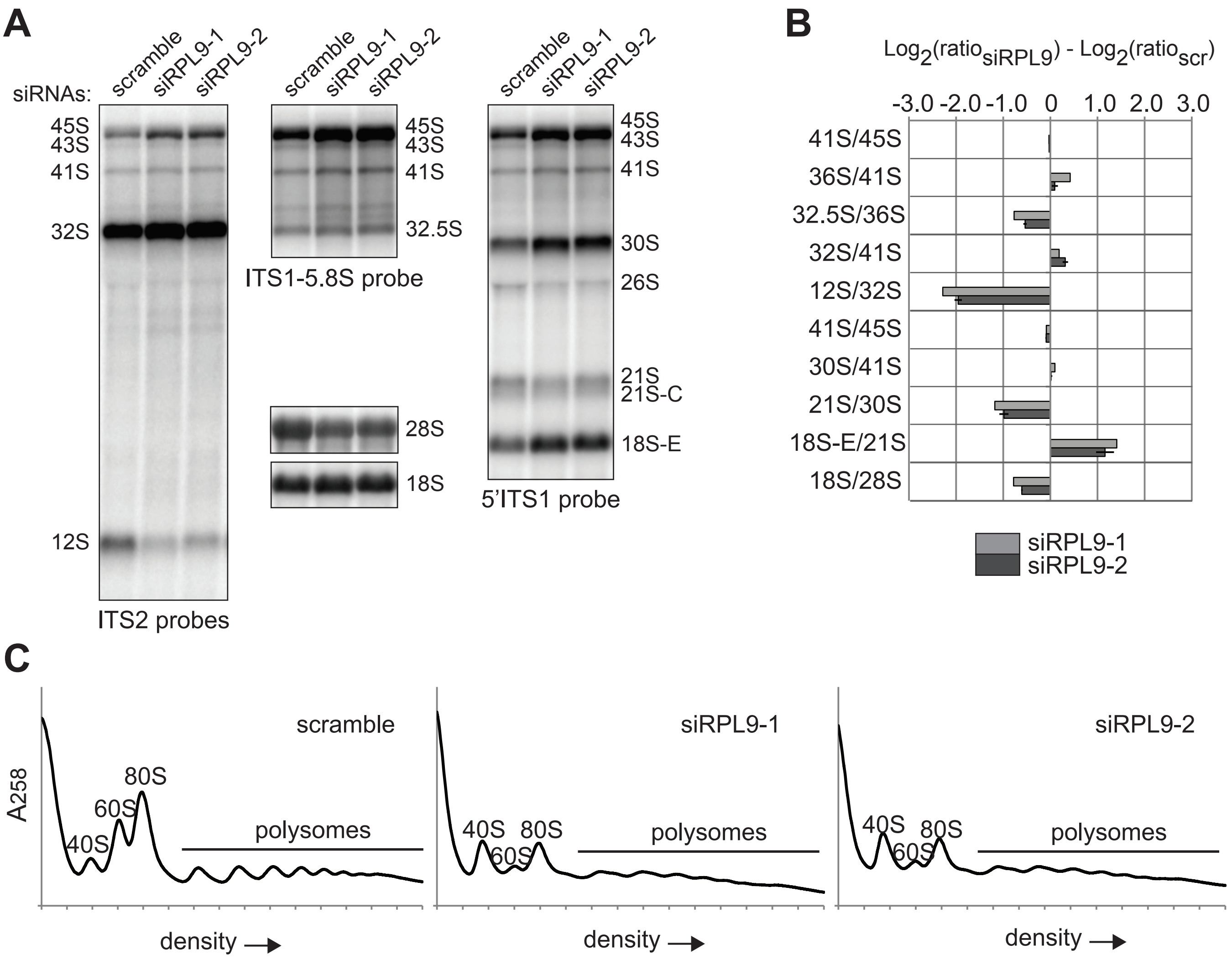

Figure S5 (related to Figures 2 and 3). Reductions of *RPL9* levels by siRNAs in HeLa cells reveal pre-rRNA processing defects and impair 60S ribosomal subunit formation.
A) Northern blot analysis of HeLa cells transfected with scrambled control siRNAs or siRNAs against *RPL9* mRNA. Probes used are against ITS2, ITS1-5.8S, and 18S-ITS1 (5'ITS1).
B) Quantitative RAMP analysis of **(A)** (three independent experiments). **C)** Polysome profile analysis of HeLa cells transfected with scrambled control siRNAs or siRNAs against *RPL9*. The small (40S) and large (60S) ribosomal subunits are labeled as well as the 80S monosome and the polysomes. Note the strong reduction of the 60S peaks in the siRPL9 samples.

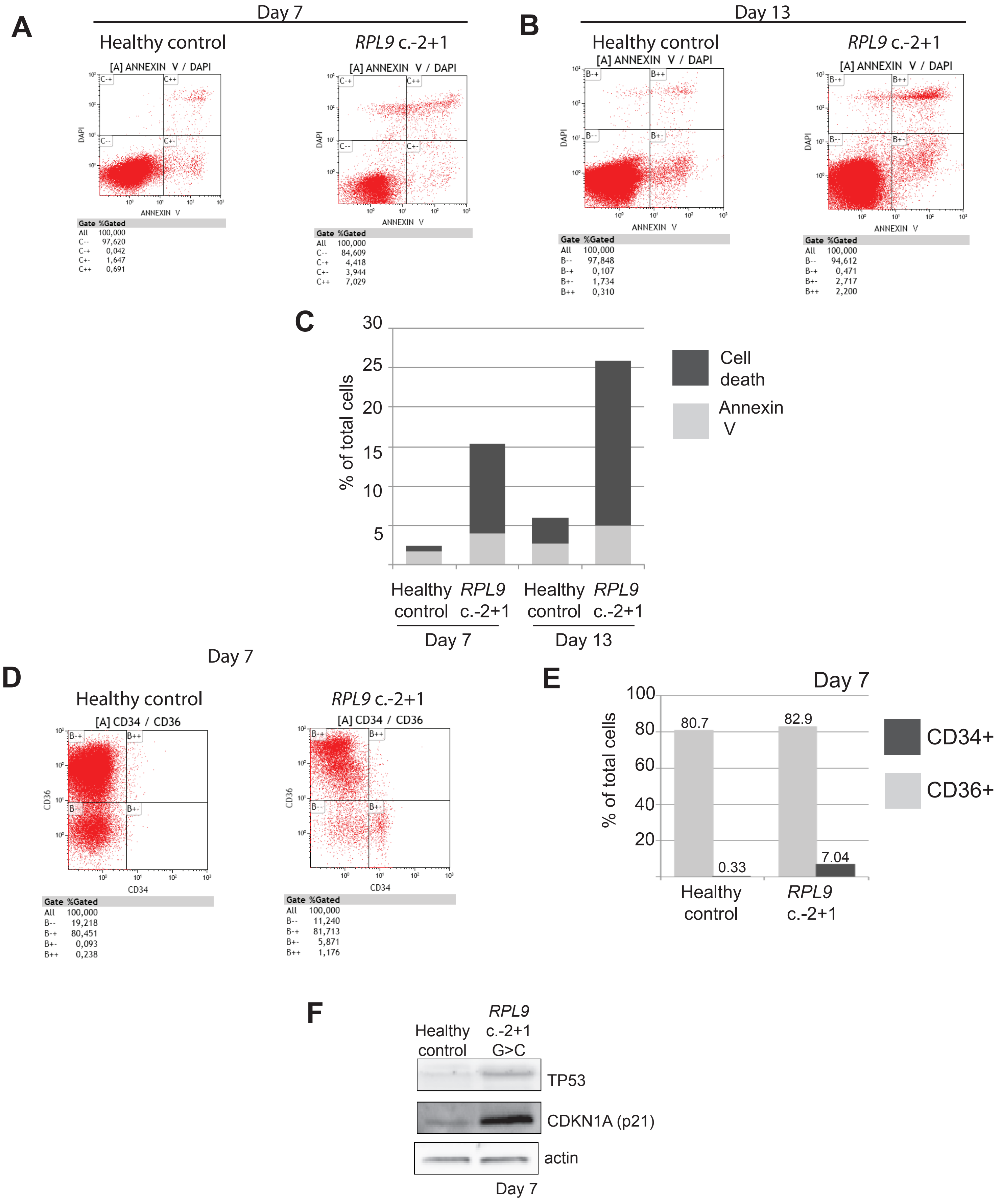

Figure S6 (Related to Figure 5). FACS plots and western blots of cells in red cell culture assays.
A-B) Day 7 (A) and Day 13 (B) Annexin V/DAPI staining of healthy control and *RPL9* c.-2+1 cells.
C) Quantification of (A) and (B). **D)** Day 7 CD34+/CD36+ staining of healthy control and *RPL9* c.-2+1 cells.
E) Quantification of (D). **F)** Western blot of cells at Day 7 with antibodies against TP53 and CDKN1A (p21).

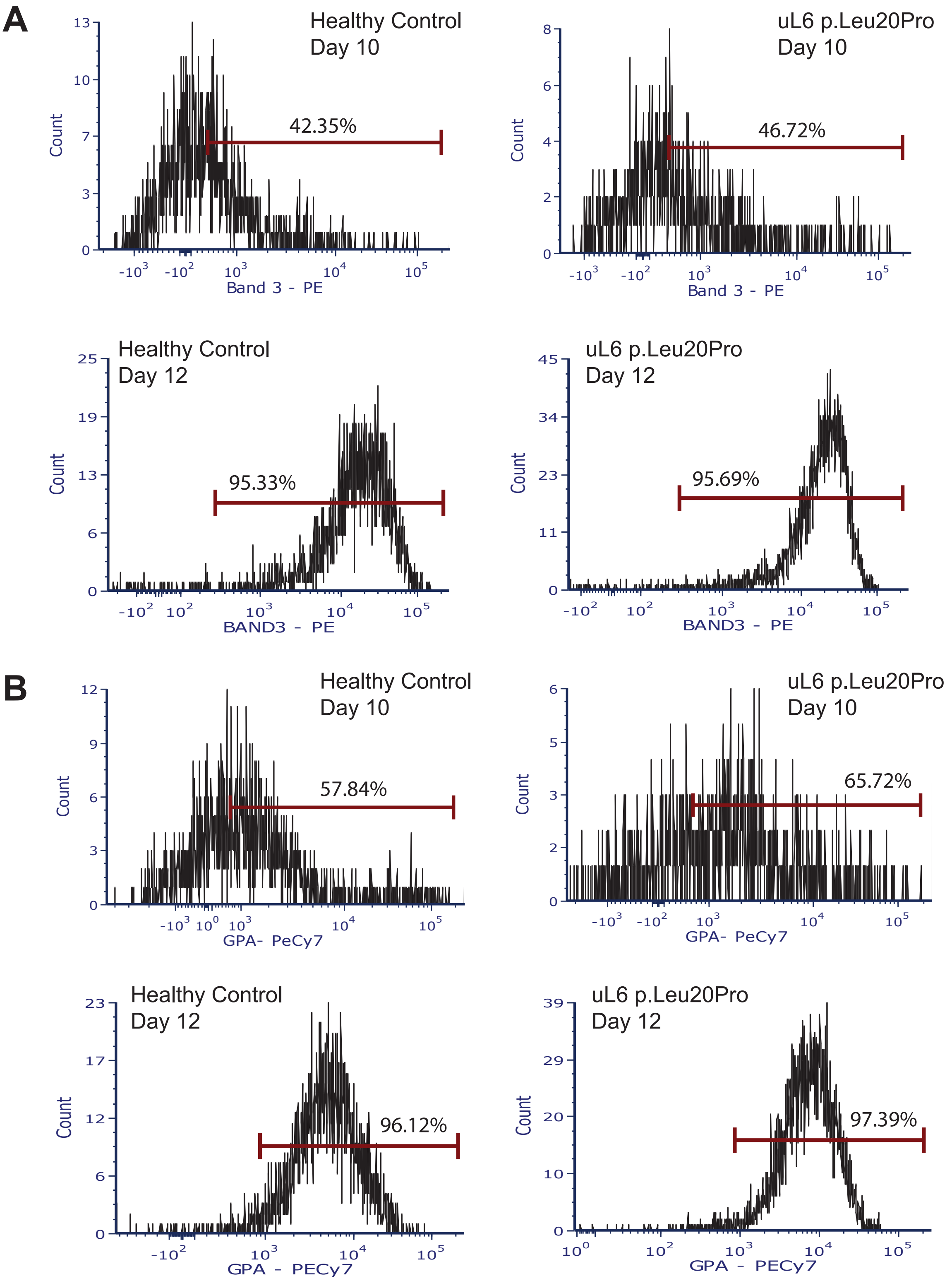

Figure S7. FACS analysis of cells derived P2 carrying the uL6 p.Leu20Pro variant compared to a healthy control in red cell culture assays. A) Cells stained with antibodies against Band3 at Days 10 and 12. **B)** Cells stained with antibodies against GPA at Days 10 and 12.

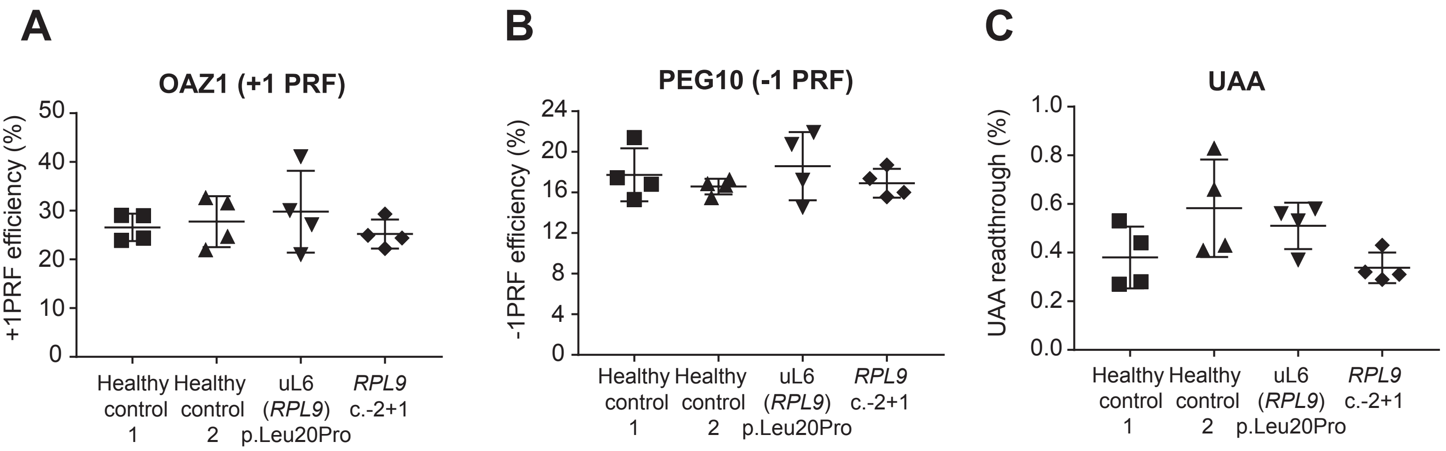

Figure S8 (related to Figure 7). Cell-based assays measuring translational fidelity. A) +1 Programmed Ribosomal Frameshifting (PRF) levels in LCLs derived from individuals carrying variants in *RPL9* compared to LCLs derived from two unrelated healthy controls. **B)** -1 PRF levels in LCLs analyzed in **(A)**. **C)** UAA stop codon read through levels in LCLs analyzed in **(A)**.
